# Supplementary material for: Clinical process quality of antenatal care in low and middle-income countries: Cross-sectional evidence from 13 countries
Source: PLOS Glob Public Health. 2026 Feb 24;6(2):e0005990. doi: 10.1371/journal.pgph.0005990 (PMC12931779; doi:10.1371/journal.pgph.0005990)
Supplement: S1 Text — This document provides additional methodological detail and supplementary results. It includes information on the Service Provision Assessment (SPA) surveys used in the analysis; sample sizes and country coverage; construction of the clinical process quality measure and the checklist items included; assessment of consultation order effects; supplementary tables and figures; and additional analyses comparing public and private facilities. (PDF) [file pgph.0005990.s001.pdf]

S1 Text. Supplementary methods and results

SPA surveys

Overview

This study uses data from all available Service Provision Assessment (SPA) surveys that contain a module on the quality of antenatal care.[1] The SPA surveys are part of the Demographic and Health Survey programme. They collect data in health facilities and focus on the availability of drugs and equipment, human resources, and the clinical quality of antenatal care. Depending on the country, additional information on the quality of care—such as family planning, child health, or other services—is collected. Quality of care measures are based on observations of medical consultations and patient exit interviews. The SPA usually include a nationally representative sample of health facilities. For every SPA survey, a report is written up which summarises the main findings. When it comes to findings on the quality of care, reports show the proportion of providers who completed each (or most) of the items included in the questionnaire. Country reports, however, do not aggregate this information into a quality score, as done here, meaning that they do not assess the overall clinical quality of care in a country. In addition, each country report focuses on slightly different indicators, meaning that it is challenging to compare findings across settings.

To illustrate the location and geographical distribution of study facilities, Fig A shows the location of sampled health facilities in the DRC and Tanzania. Additional maps for other study countries are shown in Figs B–G.[2]

[1] For countries such as Senegal that have multiple surveys collecting these data, the most recent year was used.

[2] GPS data on the location of health facilities are available for Afghanistan, DRC, Haiti, Namibia, Nepal, Senegal, Tanzania, Kenya, and Malawi.

Fig A. Location of study facilities in the DRC and Tanzania

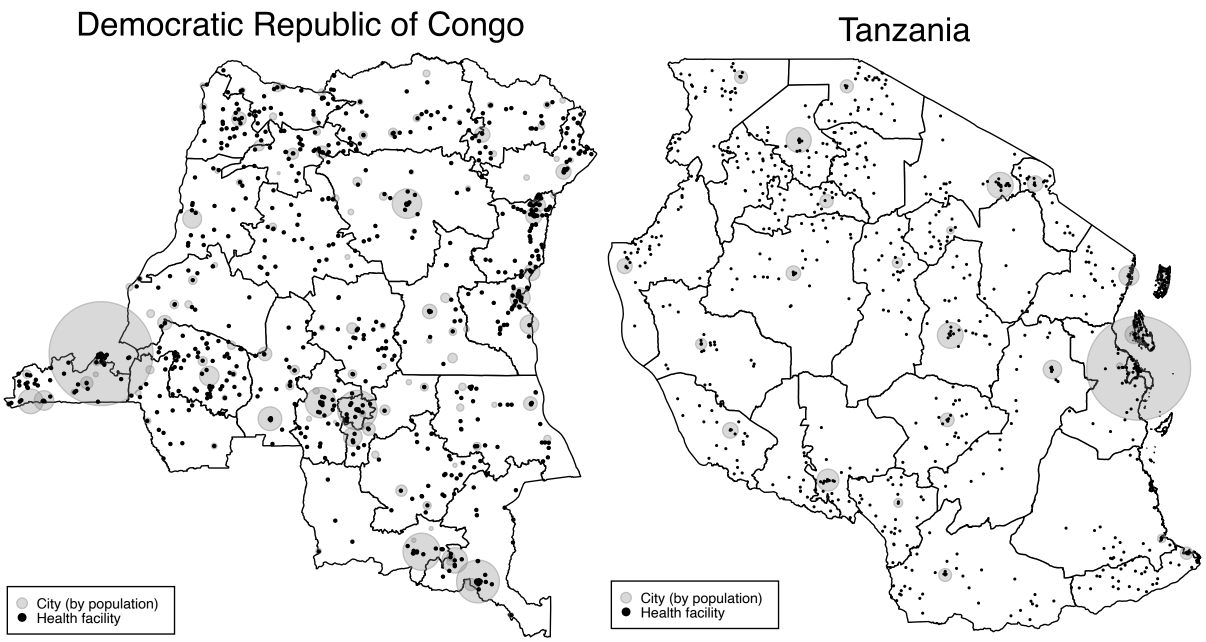

Note: The map shows the location of study facilities in the DRC and Tanzania. For reference, grey circles indicate the location and population size of the countries' main cities.

Sample sizes

As shown in Table A, sample sizes differ considerably across surveys, usually reflecting country-level differences in population size. With the exception of Afghanistan, all surveys are nationally representative.[3]

[3]The SPA in Afghanistan focuses on quality of care in seven major urban areas (Kabul, Herat, Balkh, Kandahar, Nangarhar, Kunduz, Paktya).

Table A. Sample overview

	Facilities	Providers	Consultations
Afghanistan (2018)	74	124	494
DRC (2017)	899	1,251	4,517
Egypt (2004)	320	367	1,093
Ghana (2002)	284	345	1,846
Haiti (2018)	420	526	1,528
Kenya (2010)	396	463	1,445
Malawi (2013)	412	467	2,105
Namibia (2009)	179	241	859
Nepal (2015)	464	595	1,565
Rwanda (2007)	152	152	737
Senegal (2016)	290	303	849
Tanzania (2014)	815	1,105	4,010
Uganda (2007)	207	216	802

Note: Data are based on SPA surveys. The table shows the number of health facilities sampled in each country, the number of providers who conducted antenatal consultations, and the total number of antenatal consultations observed. For all countries except Afghanistan, data are nationally representative.

Accessing SPA questionnaires

All core SPA questionnaires can be accessed via: https://dhsprogram.com/publications/publication-SPAQ8-SPA-Questionnaires-and-Manuals.cfm. As questionnaires differ slightly across study countries, the questionnaires used in each setting are shown in the final reports for each country and are available at: https://dhsprogram.com/methodology/survey-search.cfm?sendsearch=1&YrFrom=1985&YrTo=2024&str2=19,&crt=1&listgrp=0.

Location of study facilities

Fig B. Location of study facilities in Haiti

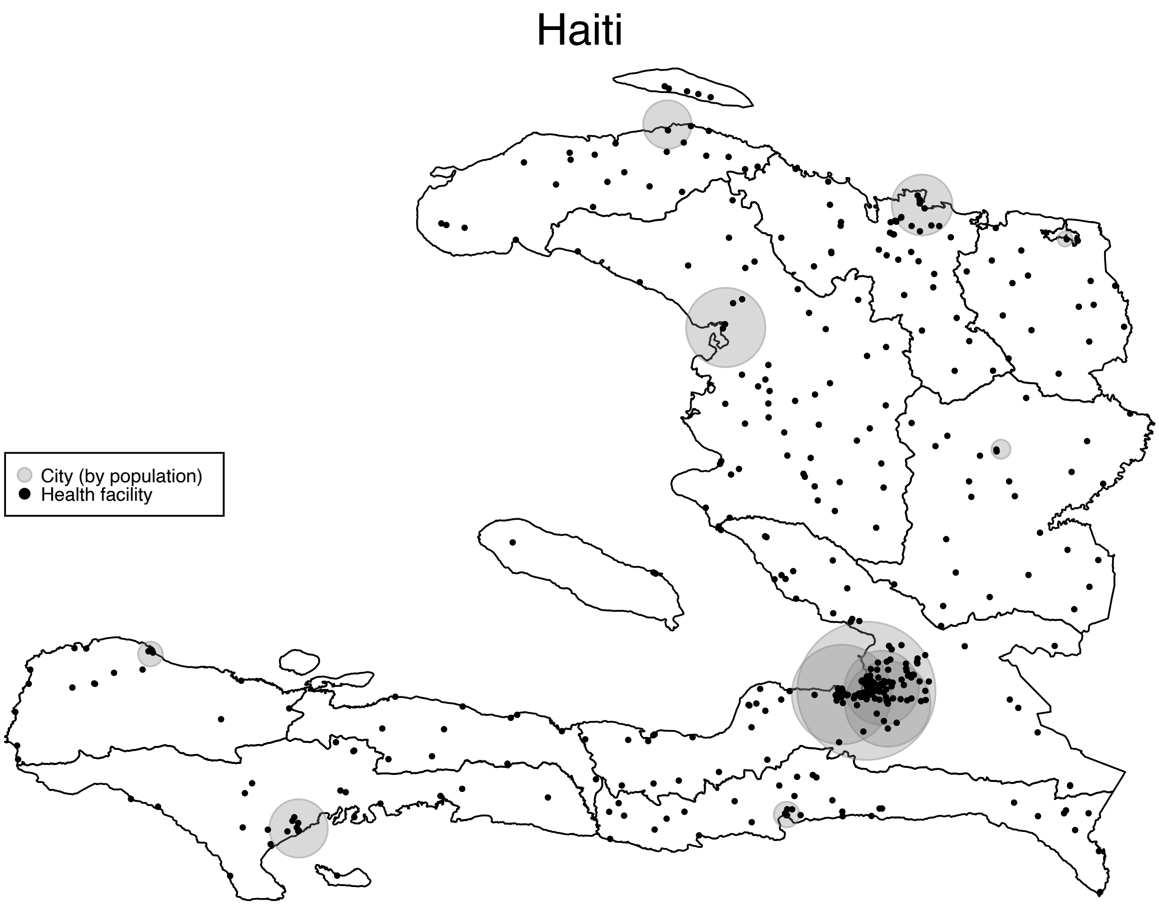

Note: Grey circles indicate the location and population size of main cities.

Fig C. Location of study facilities in Namibia

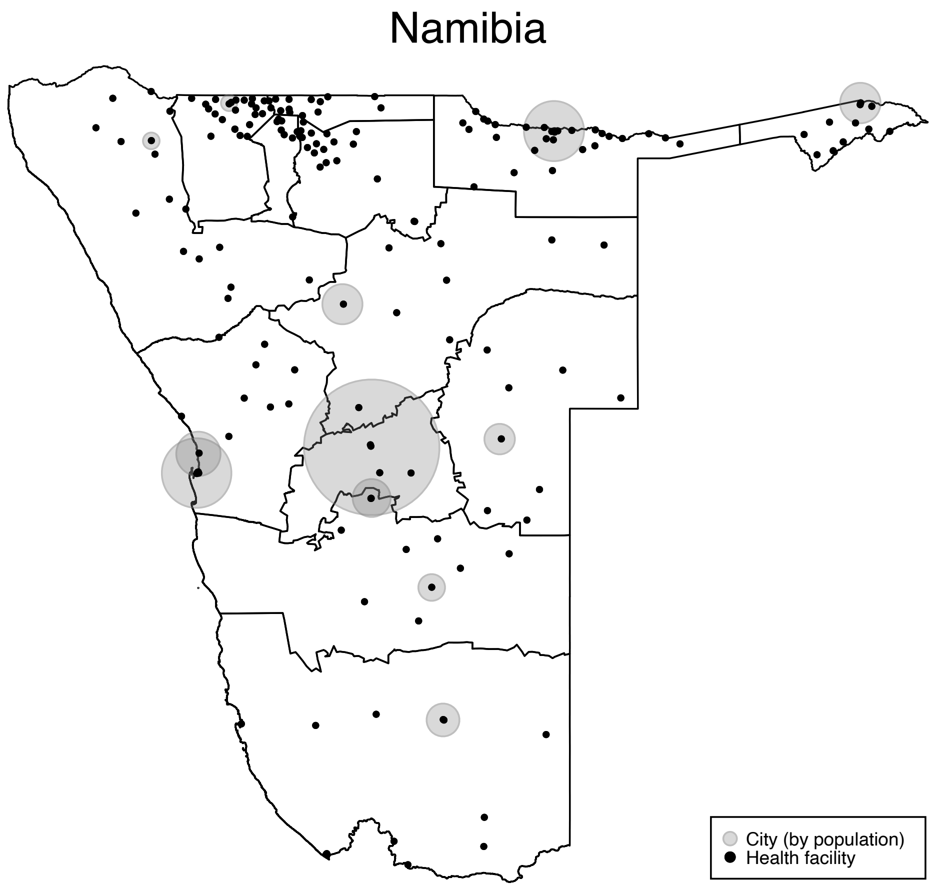

Note: Grey circles indicate the location and population size of main cities.

Fig D. Location of study facilities in Nepal

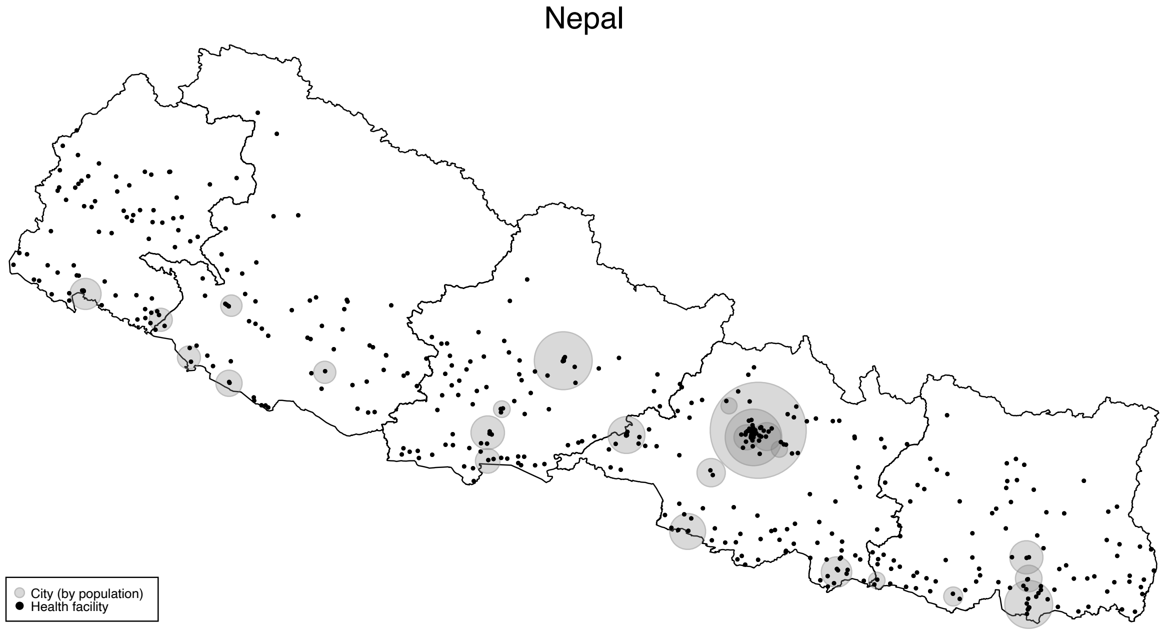

Note: Grey circles indicate the location and population size of main cities.

Fig E. Location of study facilities in Senegal

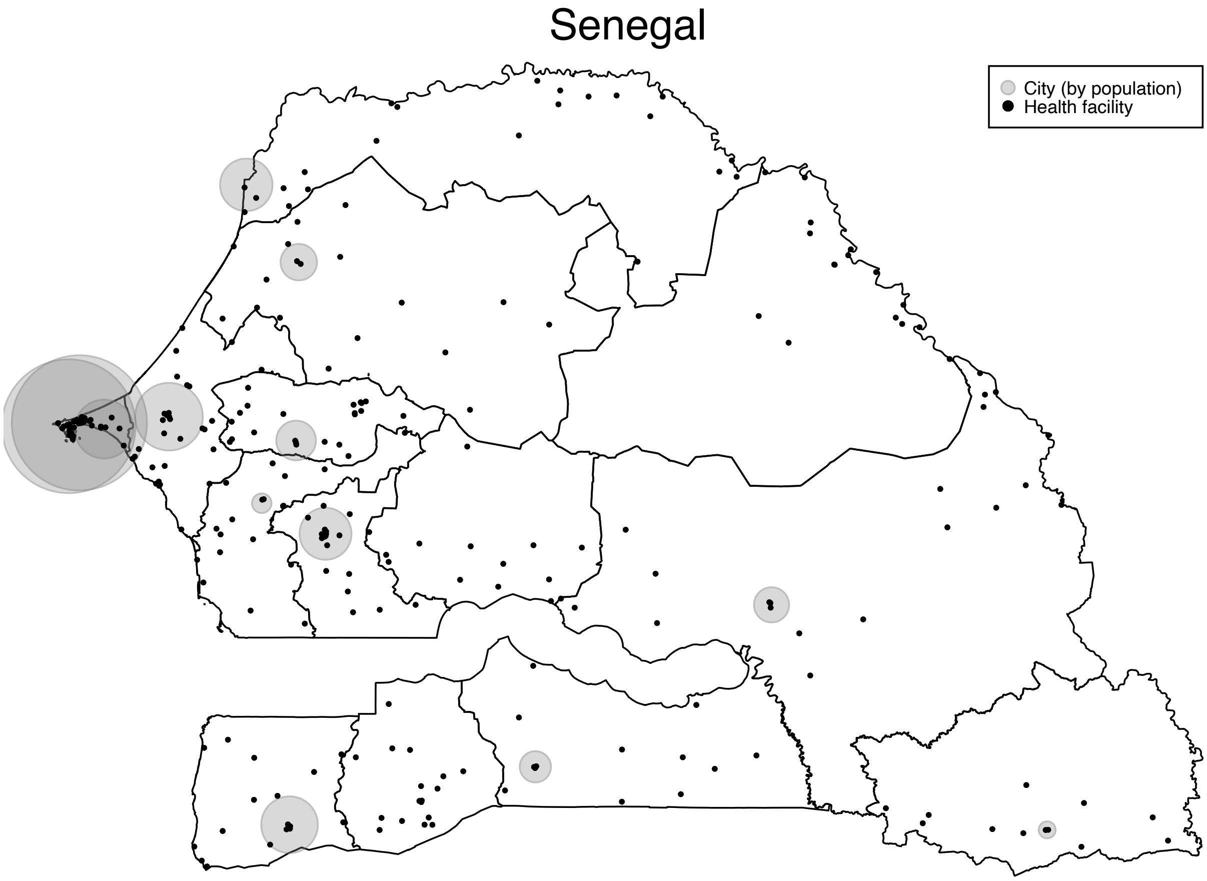

Note: Grey circles indicate the location and population size of main cities.

Fig F. Location of study facilities in Kenya

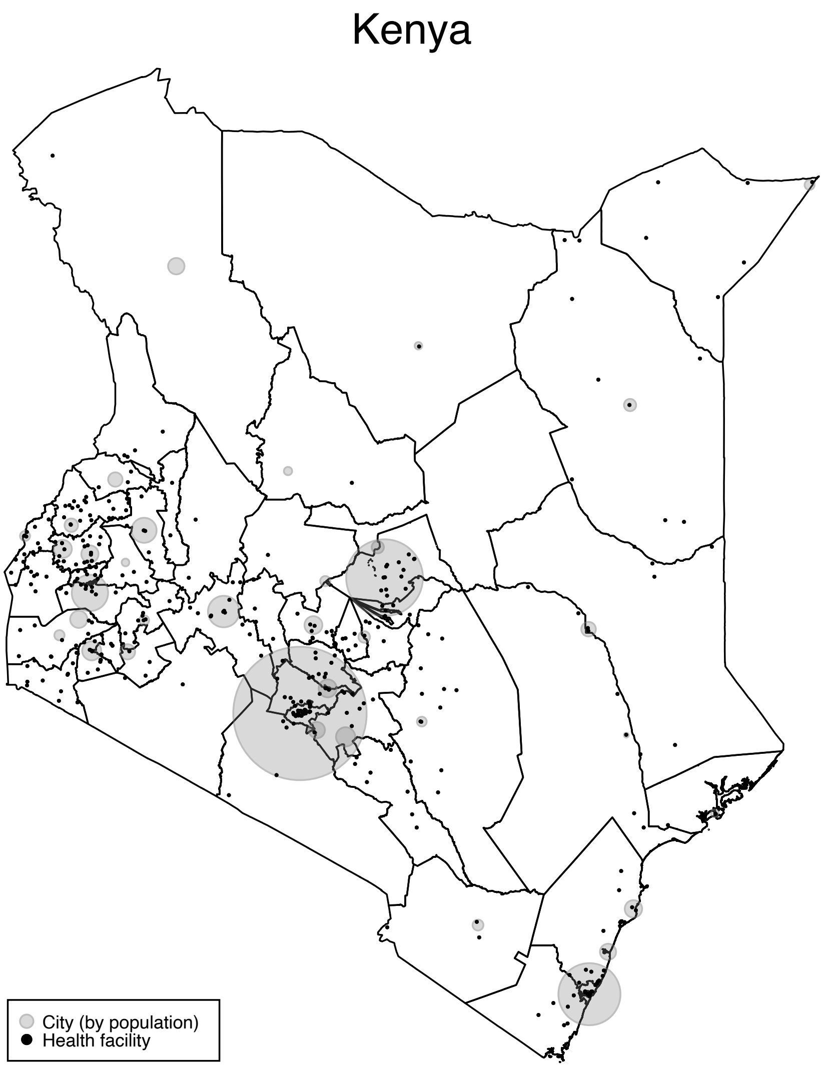

Note: Grey circles indicate the location and population size of main cities.

Fig G. Location of study facilities in Malawi

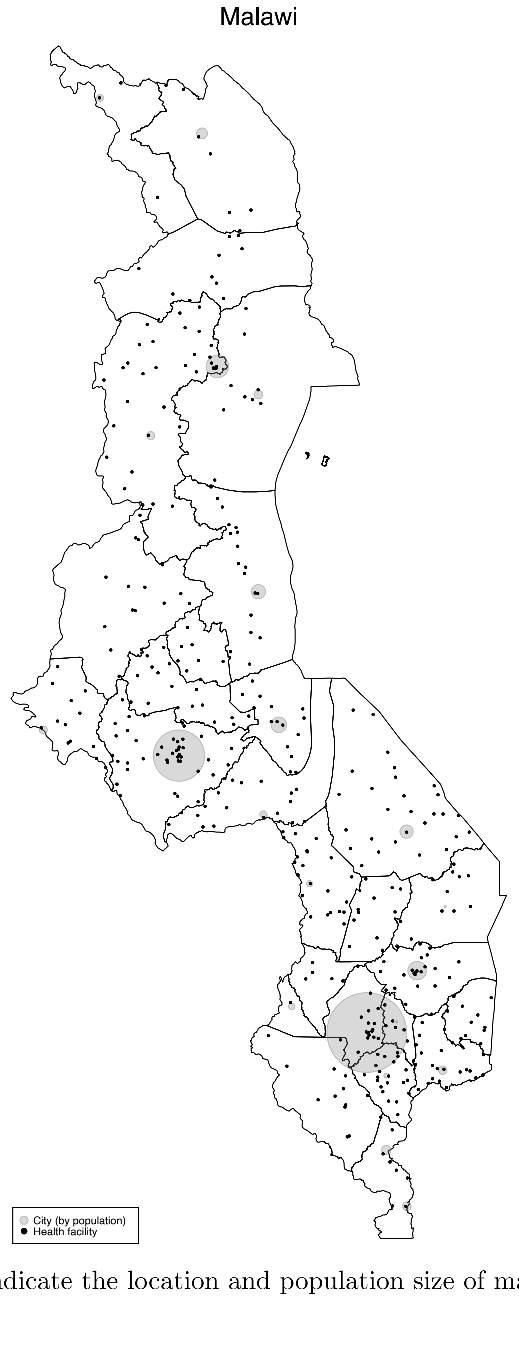

Note: Grey circles indicate the location and population size of main cities.

Order effects

One critique of clinical observations, which is based on **?**, is that quality of care likely varies considerably based on the order of consultations. **?** note that likely because of a Hawthorne effect, providers increase guideline adherence substantially for the early part of the observation period – an effect that vanishes after a handful of consultations in their data from observations of doctors in 45 facilities in Tanzania. This section examines whether quality of care decreases with the order of consultations but results do not indicate such an effect for any of the study countries, including Tanzania.

Table B. Quality of healthcare in LMICs based on the order of consultations

	(1)	(2)	(3)	(4)	(5)
VARIABLES	Full-sample	Full-sample	Full-sample	Public-sector	Private-sector
Order	0.000	-0.002*	-0.002**	-0.007***	-0.004
	(0.001)	(0.001)	(0.001)	(0.002)	(0.004)
Observations	21,850	20,372	20,326	6,578	1,582
R-squared	0.385	0.462	0.466	0.498	0.428
Facility controls		✓	✓	✓	✓
Provider controls			✓	✓	✓

Note: All models include county fixed effects. The first three columns include the full sample of 13 countries. Columns 5 and 6 only include the five countries for which both public and private-sector facilities were sampled. Robust standard errors in parentheses.

Table C. Quality of healthcare based on the order of consultations: Afghanistan

VARIABLES	(1)<br>Quality	(2)<br>Quality	(3)<br>Qualiy
Order	-0.004	-0.003	-0.003
	(0.004)	(0.004)	(0.004)
First ANC		0.033**	0.033**
		(0.015)	(0.015)
Consutlation starts before noon		0.030*	0.030*
		(0.016)	(0.016)
Observations	494	494	494
R-squared	0.747	0.755	0.755
Facility controls		✓	✓
Provider controls			✓

Note: Column 1 does not include any control variables. Column 2 controls for facility characteristics. Column 3 controls for facility and provider characteristics. Robust standard errors in parentheses.

Table D. Quality of healthcare based on the order of consultations: DRC

VARIABLES	(1)<br>Quality	(2)<br>Quality	(3)<br>Qualiy
Order	0.001	0.001	0.001
	(0.001)	(0.001)	(0.001)
First ANC		0.129***	0.129***
		(0.006)	(0.006)
Consutlation starts before noon		0.009	0.009
		(0.006)	(0.006)
Observations	4,517	4,517	4,517
R-squared	0.723	0.799	0.799
Facility controls		✓	✓
Provider controls			✓

Note: Column 1 does not include any control variables. Column 2 controls for facility characteristics. Column 3 controls for facility and provider characteristics. Robust standard errors in parentheses.

Table E. Quality of healthcare based on the order of consultations: Egypt

VARIABLES	(1)<br>Quality	(2)<br>Quality	(3)<br>Qualiy
Order	-0.001	-0.005	-0.005
	(0.004)	(0.004)	(0.004)
First ANC		0.049***	0.049***
		(0.015)	(0.015)
Consutlation starts before noon		-0.039**	-0.039**
		(0.018)	(0.018)
Observations	1,093	1,062	1,062
R-squared	0.782	0.793	0.793
Facility controls		✓	✓
Provider controls			✓

Note: Column 1 does not include any control variables. Column 2 controls for facility characteristics. Column 3 controls for facility and provider characteristics. Robust standard errors in parentheses.

Table F. Quality of healthcare based on the order of consultations: Ghana

VARIABLES	(1)<br>Quality	(2)<br>Quality	(3)<br>Qualiy
Order	0.001	-0.000	-0.000
	(0.001)	(0.002)	(0.002)
First ANC		-0.071***	-0.071***
		(0.012)	(0.012)
Consutlation starts before noon		-0.013	-0.013
		(0.012)	(0.012)
Skilled provider			-0.065
			(0.117)
Observations	1,846	1,525	1,525
R-squared	0.544	0.574	0.574
Facility controls		✓	✓
Provider controls			✓

Note: Column 1 does not include any control variables. Column 2 controls for facility characteristics. Column 3 controls for facility and provider characteristics. Robust standard errors in parentheses.

Table G. Quality of healthcare based on the order of consultations: Haiti

VARIABLES	(1)<br>Quality	(2)<br>Quality	(3)<br>Qualiy
Order	0.002	0.002	0.002
	(0.003)	(0.003)	(0.003)
First ANC		0.072***	0.072***
		(0.010)	(0.010)
Consutlation starts before noon		0.028**	0.028**
		(0.011)	(0.011)
Observations	1,528	1,528	1,528
R-squared	0.630	0.673	0.673
Facility controls		✓	✓
Provider controls			✓

Note: Column 1 does not include any control variables. Column 2 controls for facility characteristics. Column 3 controls for facility and provider characteristics. Robust standard errors in parentheses.

Table H. Quality of healthcare based on the order of consultations: Kenya

VARIABLES	(1) Quality	(2) Quality	(3) Qualiy
Order	0.001	0.000	0.000
	(0.003)	(0.003)	(0.003)
First ANC		0.115***	0.115***
		(0.010)	(0.010)
Consutlation starts before noon		0.003	0.003
		(0.011)	(0.011)
Observations	1,445	1,437	1,437
R-squared	0.743	0.802	0.802
Facility controls		✓	✓
Provider controls			✓

Note: Column 1 does not include any control variables. Column 2 controls for facility characteristics. Column 3 controls for facility and provider characteristics. Robust standard errors in parentheses.

Table I. Quality of healthcare based on the order of consultations: Malawi

VARIABLES	(1)<br>Quality	(2)<br>Quality	(3)<br>Qualiy
Order	0.002	0.001	0.001
	(0.002)	(0.001)	(0.001)
First ANC		0.099***	0.099***
		(0.009)	(0.009)
Consutlation starts before noon		0.003	0.003
		(0.012)	(0.012)
Observations	2,105	2,083	2,083
R-squared	0.754	0.792	0.792
Facility controls		✓	✓
Provider controls			✓

Note: Column 1 does not include any control variables. Column 2 controls for facility characteristics. Column 3 controls for facility and provider characteristics. Robust standard errors in parentheses.

Table J. Quality of healthcare based on the order of consultations: Namibia

VARIABLES	(1)<br>Quality	(2)<br>Quality	(3)<br>Qualiy
Order	0.001	0.002	0.002
	(0.004)	(0.004)	(0.004)
First ANC		0.130***	0.130***
		(0.037)	(0.037)
Consutlation starts before noon		-0.012	-0.012
		(0.016)	(0.016)
Observations	859	825	825
R-squared	0.821	0.836	0.836
Facility controls		✓	✓
Provider controls			✓

Note: Column 1 does not include any control variables. Column 2 controls for facility characteristics. Column 3 controls for facility and provider characteristics. Robust standard errors in parentheses.

Table K. Quality of healthcare based on the order of consultations: Nepal

VARIABLES	(1)<br>Quality	(2)<br>Quality	(3)<br>Qualiy
Order	0.002	0.002	0.002
	(0.002)	(0.002)	(0.002)
First ANC		0.052***	0.052***
		(0.008)	(0.008)
Consutlation starts before noon		-0.001	-0.001
		(0.010)	(0.010)
Observations	1,565	1,565	1,565
R-squared	0.631	0.661	0.661
Facility controls		✓	✓
Provider controls			✓

Note: Column 1 does not include any control variables. Column 2 controls for facility characteristics. Column 3 controls for facility and provider characteristics. Robust standard errors in parentheses.

Table L. Quality of healthcare based on the order of consultations: Rwanda

VARIABLES	(1)<br>Quality	(2)<br>Quality	(3)<br>Qualiy
Order	-0.002	-0.003	-0.003
	(0.003)	(0.003)	(0.003)
First ANC		0.036*	0.036*
		(0.018)	(0.018)
Consutlation starts before noon		0.002	0.003
		(0.021)	(0.021)
Observations	737	737	732
R-squared	0.863	0.865	0.866
Facility controls		✓	✓
Provider controls			✓

Note: Column 1 does not include any control variables. Column 2 controls for facility characteristics. Column 3 controls for facility and provider characteristics. Robust standard errors in parentheses.

Table M. Quality of healthcare based on the order of consultations: Senegal

VARIABLES	(1)<br>Quality	(2)<br>Quality	(3)<br>Qualiy
Order	0.000	0.002	0.002
	(0.005)	(0.005)	(0.005)
First ANC		0.132***	0.132***
		(0.014)	(0.014)
Consutlation starts before noon		-0.005	-0.004
		(0.019)	(0.019)
Skilled provider			-0.035
			(0.052)
Observations	849	849	849
R-squared	0.683	0.748	0.748
Facility controls		✓	✓
Provider controls			✓

Note: Column 1 does not include any control variables. Column 2 controls for facility characteristics. Column 3 controls for facility and provider characteristics. Robust standard errors in parentheses.

Table N. Quality of healthcare based on the order of consultations: Tanzania

VARIABLES	(1) Quality	(2) Quality	(3) Qualiy
Order	0.002	0.002	0.002
	(0.003)	(0.002)	(0.002)
First ANC		0.160***	0.160***
		(0.009)	(0.009)
Consutlation starts before noon		0.012	0.012
		(0.010)	(0.010)
Observations	4,010	4,010	4,010
R-squared	0.677	0.739	0.739
Facility controls		✓	✓
Provider controls			✓

Note: Column 1 does not include any control variables. Column 2 controls for facility characteristics. Column 3 controls for facility and provider characteristics. Robust standard errors in parentheses.

Table O. Quality of healthcare based on the order of consultations: Uganda

VARIABLES	(1) Quality	(2) Quality	(3) Qualiy
Order	-0.001	0.002	0.002
	(0.004)	(0.003)	(0.003)
First ANC		0.115***	0.122***
		(0.016)	(0.016)
Consutlation starts before noon		0.030	0.027
		(0.020)	(0.020)
Observations	802	802	761
R-squared	0.778	0.819	0.816
Facility controls		✓	✓
Provider controls			✓

Note: Column 1 does not include any control variables. Column 2 controls for facility characteristics. Column 3 controls for facility and provider characteristics. Robust standard errors in parentheses.

Items used to measure the clinical quality of antenatal care

Table P below shows the items used to capture the clinical quality of antenatal care. Importantly, these actions represent the absolute minimum standard for antenatal care, as only the most essential actions are included.

Table P. Items included in the clinical quality of care measure

**History:**‡	**Physical examinations:**
Client's age	Blood pressure
Medications the client is taking	Weight
Last menstrual period	Examine eyes/palms for anemia
Number of prior pregnancies	Examine legs/feet/hands for edema
**Previous pregnancies:**§	Examine lymphnodes
Prior stillbirths	Palpate for fetal presentation*
Deaths in first week of life	Palpate for fundal height†
Heavy bleeding	Listen for fetal heartbeat†
Previous assisted delivery	Examine the client's breasts
Previous pregnancy loss	**Tests:**‡
**Counselling:**	Hemoglobin test
Vaginal bleeding	**Preventive treatments:**
Fever	Iron or folic acid
Headache or blurred vision	
Swollen face or hands or extremities	
Tiredness or breathlessness	
Concerns about fetal movement†	
Ask about any concerns/symptoms	

Note: An ultrasound is considered a substitute for palpating the abdomen for fetal presentation, measuring fundal height, or listening to the fetal heartbeat. ‡ Only for a first consultation. § Only for women with previous pregnancies. * Only relevant from 36 weeks pregnant. †Only relevant from 20 weeks pregnant.

Table Q. Countries with missing quality-of-care items

	Namibia	Rwanda	Kenya	Ghana	Uganda	Egypt
**History:**						
Client's age						
Medications the client is taking				x		x
Last menstrual period						
Prior pregnancies						
None of the above						
**Previous pregnancies:**						
Prior stillbirth(s)						
Deaths in the first week of life						
Heavy bleeding, during or after delivery						
Previous assisted delivery						
Previous spontaneous abortions				x		
**Danger signs:**						
Vaginal bleeding						
Fever				x		x
Headache or blurred vision				x		x
Swollen face or hands or extremities				x		x
Tiredness or breathlessness				x		x
Fetal movement						
Any other symptoms or problems						
**Physical examinations:**						
Take the client's blood pressure						
Weigh the client						
Examine for anemia		x		x	x	x
Examine for edema				x	x	x
Examine lymphnodes	x					x
Fetal presentation						
Fundal height						
Fetal heartbeat						
Conduct an ultrasound	x		x		x	
Examine the client's breasts						x
**Tests:**						
Hemoglobin test						
**Prevention:**						
Iron/Folic						

Note: A cross indicates missing items in a given survey.

Structural quality

Fig H. Structural quality by country

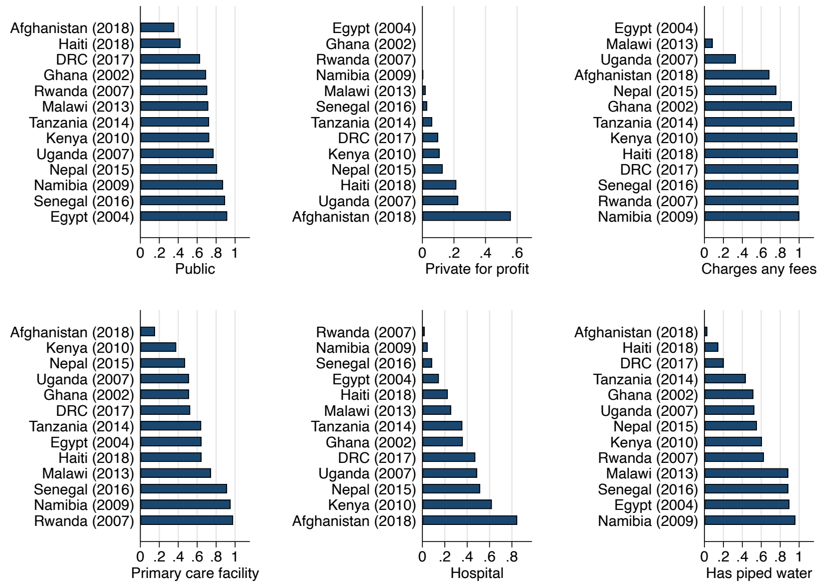

Note: The bar charts show unadjusted country-level means.

Fig I. Structural quality by country

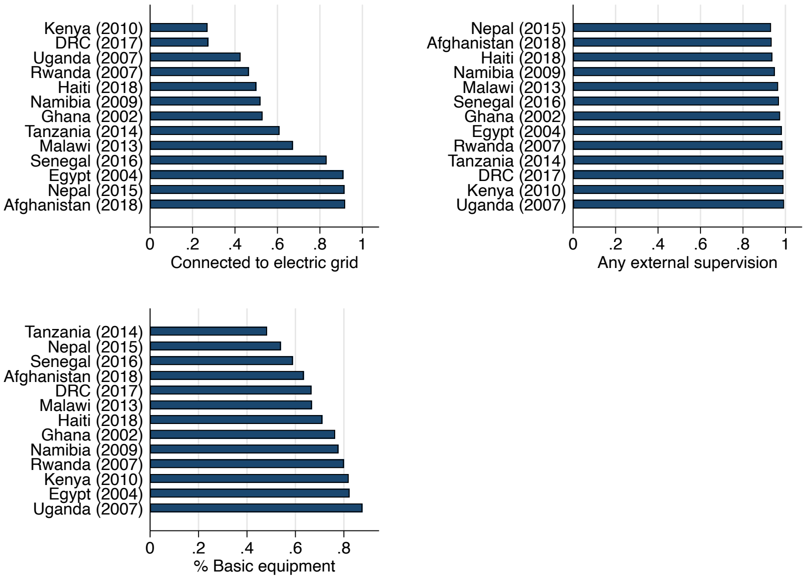

Note: The bar charts show unadjusted country-level means.

Table R. Sample description (facilities)

	(1) Afghanistan		(2) DRC		(3) Egypt		(4) Ghana		(5) Haiti		(6) Kenya		(7) Malawi	
	mean	sd	mean	sd	mean	sd	mean	sd	mean	sd	mean	sd	mean	sd
Public	0.20	0.40	0.62	0.49	0.89	0.31	0.58	0.49	0.38	0.49	0.67	0.47	0.67	0.47
Private for profit	0.68	0.47	0.11	0.32	.	.	.	.	0.26	0.44	0.16	0.37	0.04	0.19
Non-profit	0.12	0.33	0.27	0.44	0.00	0.00	0.00	0.00	0.36	0.48	0.16	0.37	0.30	0.46
Charges any fees	0.82	0.38	0.99	0.10	.	.	0.89	0.31	0.99	0.11	0.98	0.15	0.10	0.31
Below primary care	0.00	0.00	0.00	0.00	0.24	0.43	0.00	0.00	0.22	0.41	0.00	0.00	0.00	0.00
Primary care facility	0.27	0.45	0.55	0.50	0.63	0.48	0.56	0.50	0.65	0.48	0.48	0.50	0.78	0.41
Hospital	0.73	0.45	0.45	0.50	0.13	0.33	0.19	0.40	0.13	0.34	0.52	0.50	0.22	0.41
Has piped water	0.01	0.12	0.17	0.37	0.88	0.32	0.48	0.50	0.13	0.33	0.58	0.49	0.88	0.32
Connected to electric grid	0.95	0.23	0.23	0.42	0.91	0.29	0.48	0.50	0.43	0.50	0.31	0.46	0.66	0.48
Any external supervision	0.95	0.23	0.99	0.10	0.97	0.16	0.95	0.21	0.93	0.26	0.99	0.10	0.96	0.19
% basic equipment	0.61	0.26	0.67	0.18	0.80	0.18	0.76	0.16	0.69	0.19	0.81	0.15	0.68	0.20
Number of skilled medical staff	71.26	110.84	25.66	75.64	.	.	10.24	26.34	9.74	10.83	7.73	8.78	0.81	1.76
% Doctors	0.48	0.11	0.14	0.15	.	.	0.07	0.17	0.41	0.33	0.06	0.11	0.11	0.24
% Nurses	0.32	0.15	0.84	0.16	.	.	0.47	0.08	0.35	0.20	0.74	0.21	0.85	0.29
% Midwives	0.20	0.11	0.02	0.05	.	.	0.47	0.08	0.24	0.17	0.21	0.19	0.04	0.18
Outpatient visits in past month	4422.13	8377.51	272.43	362.13	.	.	.	.	776.33	1195.44	1923.63	2833.57	1892.79	1656.43
Visits per skilled staff per day	3.66	5.36	1.63	2.46	.	.	.	.	4.53	8.92	11.92	19.12	63.38	59.74
Observations	74		899		320		284		420		396		412	

Note: Data are based on SPA surveys.

Table S. Sample description (facilities), continued

	(1) Namibia		(2) Nepal		(3) Rwanda		(4) Senegal		(5) Tanzania		(6) Uganda	
	mean	sd	mean	sd	mean	sd	mean	sd	mean	sd	mean	sd
Public	0.88	0.33	0.84	0.37	0.70	0.46	0.88	0.32	0.73	0.44	0.77	0.42
Private for profit	0.02	0.13	0.11	0.32	.	.	0.03	0.18	0.07	0.26	0.23	0.42
Non-profit	0.11	0.31	0.05	0.21	0.00	0.00	0.08	0.28	0.19	0.39	0.00	0.00
Charges any fees	1.00	0.00	0.64	0.48	0.99	0.08	0.99	0.10	0.94	0.23	0.32	0.47
Below primary care	0.00	0.00	0.01	0.10	0.00	0.00	0.00	0.00	0.00	0.00	0.00	0.00
Primary care facility	0.97	0.17	0.65	0.48	0.97	0.16	0.92	0.27	0.73	0.44	0.59	0.49
Hospital	0.03	0.17	0.34	0.47	0.03	0.16	0.08	0.27	0.27	0.44	0.41	0.49
Has piped water	0.96	0.19	0.48	0.50	0.62	0.49	0.83	0.38	0.40	0.49	0.47	0.50
Connected to electric grid	0.47	0.50	0.86	0.35	0.47	0.50	0.75	0.43	0.55	0.50	0.38	0.49
Any external supervision	0.94	0.23	0.95	0.23	0.98	0.14	0.96	0.20	0.99	0.12	1.00	0.07
% basic equipment	0.78	0.21	0.56	0.20	0.80	0.17	0.61	0.16	0.46	0.29	0.86	0.15
Number of skilled medical staff	97.52	644.33	4.47	5.37	13.24	24.91	7.89	24.09	3.76	5.23	15.85	50.34
% Doctors	0.02	0.08	0.07	0.16	0.02	0.06	0.08	0.15	0.06	0.13	0.05	0.11
% Nurses	0.98	0.08	0.79	0.24	0.97	0.10	0.43	0.30	0.51	0.35	0.31	0.25
% Midwives	.	.	.	.	0.01	0.08	0.49	0.31	.	.	0.64	0.28
Outpatient visits in past month	737.63	1286.92	1893.04	4515.81	.	.	598.20	782.05	965.69	1531.15	.	.
Visits per skilled staff per day	7.56	28.32	17.38	21.81	.	.	7.88	8.78	12.32	13.04	.	.
Observations	179		464		152		290		815		207	

Note: Data are based on SPA surveys.

Provider characteristics

Fig J. Provider characteristics by country

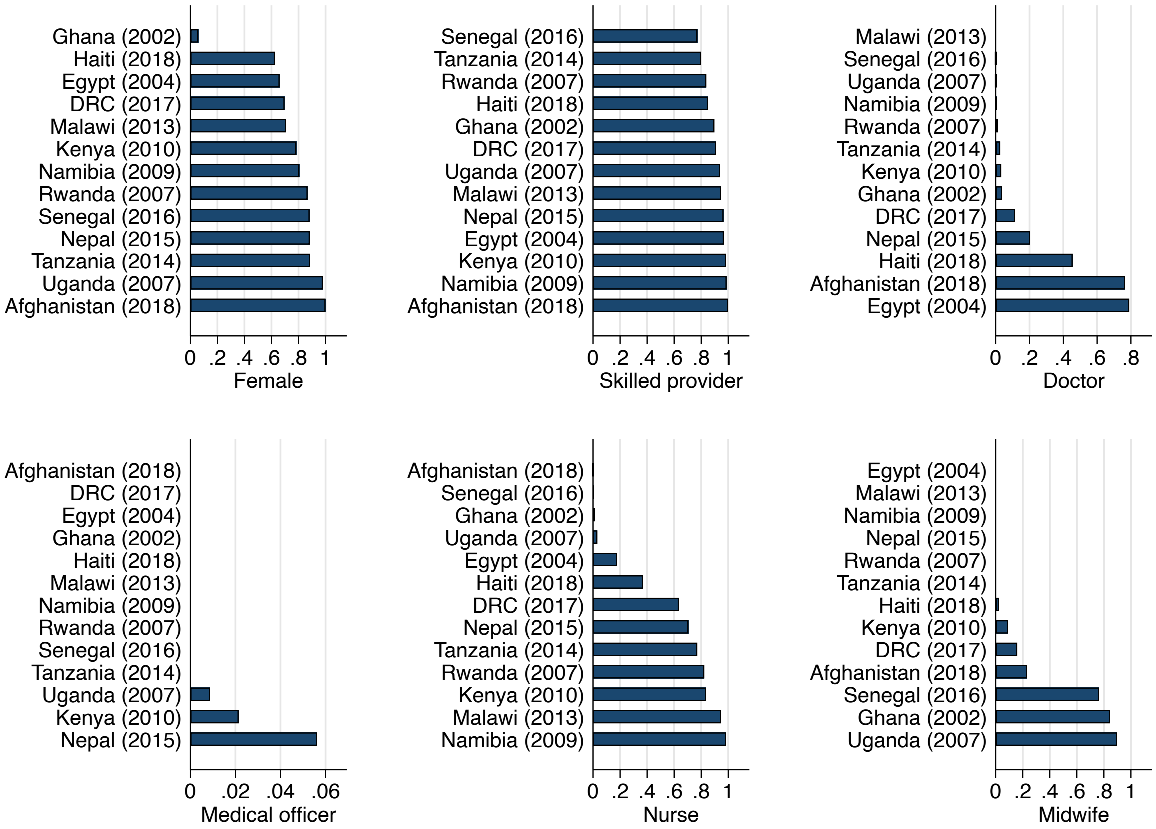

Note: The bar charts show unadjusted country-level means.

Fig K. Provider characteristics by country

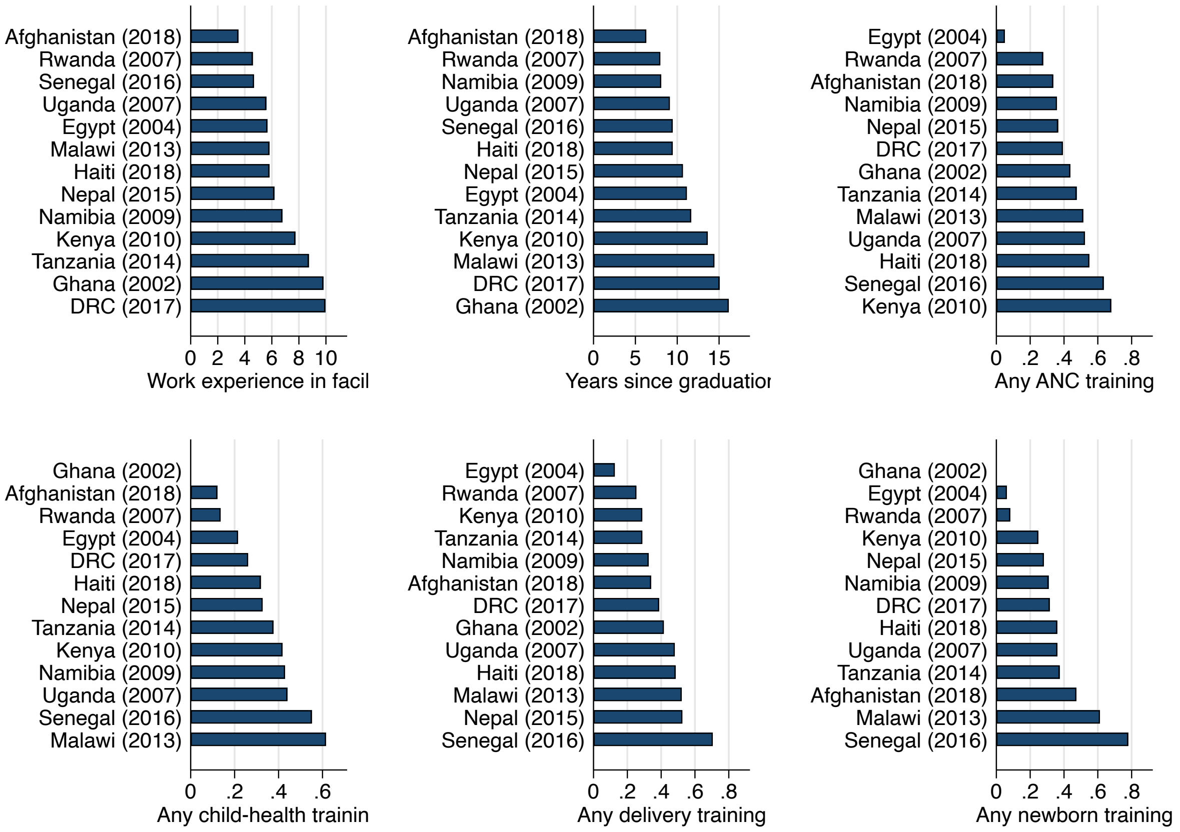

Note: The bar charts show unadjusted country-level means.

Fig L. Provider characteristics by country

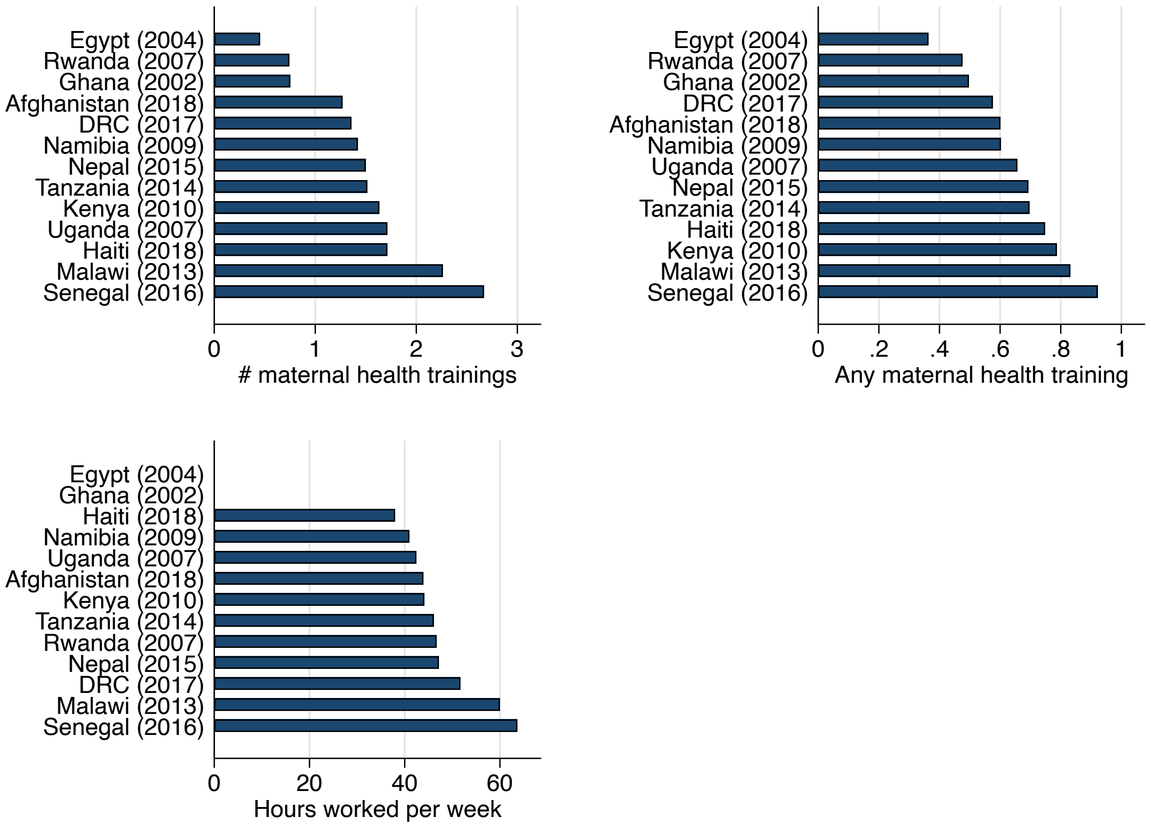

Note: The bar charts show unadjusted country-level means.

Table T. Sample description (providers)

	(1) Afghanistan		(2) DRC		(3) Egypt		(4) Ghana		(5) Haiti		(6) Kenya		(7) Malawi	
	mean	sd	mean	sd	mean	sd	mean	sd	mean	sd	mean	sd	mean	sd
Female	1.00	0.00	0.67	0.47	0.63	0.48	0.07	0.25	0.62	0.49	0.76	0.43	0.71	0.45
Skilled provider	1.00	0.00	0.91	0.29	0.98	0.15	0.86	0.35	0.83	0.38	0.98	0.13	0.94	0.23
Doctor	0.74	0.44	0.13	0.34	0.80	0.40	0.04	0.20	0.48	0.50	0.03	0.17	0.00	0.00
Medical officer	.	.	.	.	.	.	.	.	.	.	0.03	0.18	.	.
Nurse	0.01	0.09	0.63	0.48	0.18	0.38	0.02	0.14	0.33	0.47	0.83	0.38	0.94	0.23
Midwife	0.25	0.43	0.15	0.36	.	.	0.80	0.40	0.02	0.14	0.09	0.29	.	.
Work experience in facility	3.45	3.73	9.58	9.70	5.67	6.19	8.89	9.08	5.90	7.17	7.39	7.78	6.07	7.74
Years since graduation	6.27	6.14	14.76	11.74	11.23	8.01	17.42	11.76	9.91	9.44	13.48	10.37	14.60	14.65
Any ANC training	0.34	0.48	0.38	0.49	0.04	0.20	0.43	0.50	0.51	0.50	0.68	0.47	0.51	0.50
Any child-health training	0.10	0.31	0.26	0.44	0.22	0.41	.	.	0.36	0.48	0.43	0.50	0.60	0.49
Any delivery training	0.31	0.47	0.37	0.48	0.13	0.34	0.43	0.50	0.44	0.50	0.31	0.46	0.51	0.50
Any newborn training	0.44	0.50	0.30	0.46	0.08	0.27	.	.	0.32	0.47	0.26	0.44	0.60	0.49
Trainings related to maternal/newborn health	1.19	1.22	1.32	1.44	0.47	0.70	0.75	0.86	1.63	1.38	1.68	1.29	2.22	1.46
Any training on maternal/newborn health	0.60	0.49	0.56	0.50	0.37	0.48	0.48	0.50	0.72	0.45	0.79	0.41	0.82	0.38
Hours worked per week	43.90	16.83	51.91	13.51	.	.	.	.	37.21	15.08	44.33	10.98	59.39	21.75
Observations	124		1251		367		345		526		463		467	

Note: Data are based on SPA surveys.

Table U. Sample description (providers), continued

	(1) Namibia		(2) Nepal		(3) Rwanda		(4) Senegal		(5) Tanzania		(6) Uganda	
	mean	sd	mean	sd	mean	sd	mean	sd	mean	sd	mean	sd
Female	0.80	0.40	0.88	0.33	0.87	0.34	0.84	0.37	0.89	0.32	0.96	0.19
Skilled provider	0.99	0.09	0.94	0.24	0.85	0.36	0.71	0.45	0.78	0.41	0.93	0.25
Doctor	0.01	0.09	0.17	0.37	0.02	0.14	0.00	0.06	0.03	0.16	0.00	0.07
Medical officer	.	.	0.05	0.22	.	.	.	.	.	.	0.03	0.18
Nurse	0.98	0.13	0.72	0.45	0.83	0.38	0.02	0.13	0.75	0.43	0.06	0.23
Midwife	.	.	.	.	.	.	0.69	0.46	.	.	0.84	0.37
Work experience in facility	6.87	8.73	5.88	6.95	4.45	5.08	4.53	6.30	8.28	9.67	5.74	6.74
Years since graduation	8.31	8.09	10.79	8.82	7.68	7.15	9.32	7.98	11.73	10.86	9.14	9.09
Any ANC training	0.34	0.48	0.38	0.49	0.27	0.45	0.63	0.48	0.46	0.50	0.52	0.50
Any child-health training	0.42	0.50	0.36	0.48	0.11	0.31	0.62	0.49	0.38	0.49	0.46	0.50
Any delivery training	0.30	0.46	0.54	0.50	0.26	0.44	0.68	0.47	0.29	0.45	0.46	0.50
Any newborn training	0.29	0.46	0.31	0.46	0.08	0.27	0.77	0.42	0.38	0.49	0.35	0.48
Trainings related to maternal/newborn health	1.36	1.48	1.59	1.34	0.71	0.94	2.70	1.33	1.52	1.34	1.69	1.54
Any training on maternal/newborn health	0.59	0.49	0.72	0.45	0.46	0.50	0.92	0.27	0.70	0.46	0.66	0.47
Hours worked per week	41.06	3.92	47.83	23.95	47.03	9.28	65.30	19.57	46.02	10.27	43.65	11.80
Observations	241		595		152		303		1105		216	

Note: Data are based on SPA surveys.

Workload

To calculate the number of patients seen per provider per day, we compare the total number of visits to the number of skilled staff. We assume 20 working days per month and an equal distribution of patients across skilled providers.

The assumption that a given consultation lasts 15 minutes is generated based on previous studies using standardised patients, which provide high-quality evidence on the duration of medical consultations. Studies in India suggest that interactions between patients and

providers are very short and last well under five minutes, for example (?). Studies in African countries usually find slightly longer consultation times; for instance, (?) find that consultations in public facilities in Senegal lasted 13 minutes on average, and (?) find that providers in Kenya spent between 5 and 10 minutes depending on the case.

The quality of healthcare

Fig M. Average quality of antenatal care in LMICs

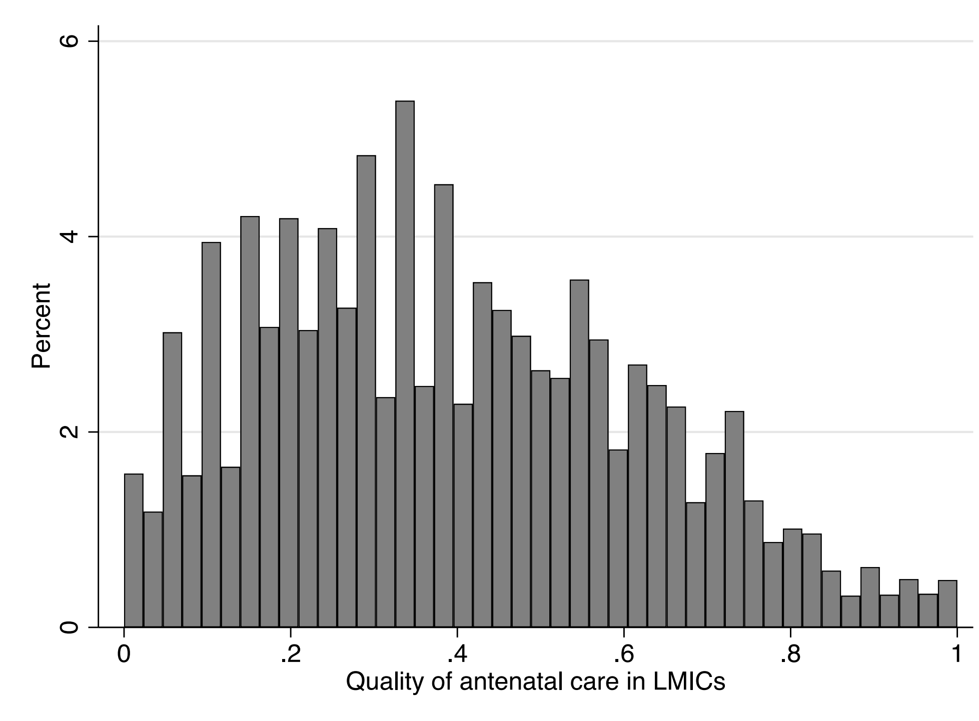

Note: The figure shows the distribution of patient-level quality of antenatal care in 13 LMICs (Afghanistan, DRC, Egypt, Ghana, Haiti, Kenya, Malawi, Namibia, Nepal, Rwanda, Senegal, Tanzania, and Uganda).

Quality of care in the private sector

To examine whether the quality of care is higher in the public compared with the private for-profit sector, we estimate OLS regression models:

$$Y_{ifpc} = \alpha F_f + \beta P_p + \gamma PV_f + \delta \left(PV_f \times \eta_c\right) + \eta_c + \epsilon_{ifpc}. \quad (1)$$

Here, Y_{ifpc} is the quality of healthcare supplied to patient i in facility f by provider p in country c (proportion of relevant history questions asked, physical examinations completed, and tests prescribed). Models include facility and provider characteristics (F_f and P_p) as well as country fixed effects η_c. PV_f indicates whether a facility is private for-profit (rather than public or not-for-profit), and $PV_f \times \eta_c$ allows this association to vary by country. Only countries where both public and private facilities were sampled are included (DRC, Haiti, Kenya, Nepal, and Uganda). As sample sizes vary across countries, we apply a weight equal to $\frac{1{,}000}{N}$, where N is the number of observed consultations in a given country, reflecting that roughly 1,000 antenatal consultations were observed in most settings.

Tables V and W provide an overview of provider and facility characteristics in the public and private sectors. Private-sector facilities are more likely to be hospitals and have higher infrastructural quality, as they are more likely to have piped water and electricity. Private facilities also have a higher availability of basic equipment (73% compared with 68%), and the proportion of doctors working in private facilities is higher (27% compared with 13%). Among providers observed during antenatal care consultations, the share of female providers is lower in the private sector, whilst the share of skilled providers observed is higher (in particular doctors, who account for 39% of consultations in the private sector and 14% in the public sector).[4] Private-sector providers have somewhat less experience (11 years since graduation compared with 13 years in the public sector), but there are no differences in on-the-job training. Private-sector providers report fewer weekly working hours (44 hours) compared with those in the public and non-profit sector (47 hours).

[4] A number of influential studies on the quality of care in LMICs were conducted in India, where private-sector providers are oftentimes unskilled and unlicensed (**?**). The data presented here indicate that this is not the case for all LMICs.

Table V. Facility characteristics in the private (for-profit) and public/non-profit (NP) sector

	Private for-profit		Public and NP		
	mean	sd	mean	sd	p
Below primary	0.05	0.22	0.04	0.19	0.29
Primary	0.49	0.50	0.59	0.49	0.00
Hospital	0.46	0.50	0.37	0.48	0.00
Has piped water	0.40	0.49	0.30	0.46	0.00
Connected to electric grid	0.52	0.50	0.39	0.49	0.00
Any external supervision	0.94	0.23	0.98	0.16	0.00
% basic equipment	0.73	0.20	0.68	0.20	0.00
Number of skilled medical staff	10.48	12.76	15.73	54.03	0.06
% Doctors	0.27	0.26	0.13	0.21	0.00
% Nurses	0.54	0.27	0.71	0.28	0.00
% Midwives	0.19	0.23	0.16	0.24	0.12
Outpatient visits in past month	1,178.34	3,545.08	858.10	2,181.67	0.04
Visits per skilled staff per day	6.27	18.90	6.61	12.95	0.71
N	375		2,011		

Note: Data are shown for countries where both public and private facilities were sampled (DRC, Haiti, Kenya, Nepal, and Uganda).

Table W. Provider characteristics in the private (for-profit) and public/non-profit (NP) sector

	Private for-profit		Public and NP		
	mean	sd	mean	sd	p
Female	0.61	0.49	0.76	0.43	0.00
Skilled provider	0.95	0.22	0.91	0.29	0.01
Doctor	0.39	0.49	0.14	0.34	0.00
Medical officer	0.12	0.33	0.03	0.16	0.00
Nurse	0.36	0.48	0.62	0.49	0.00
Midwife	0.17	0.38	0.17	0.38	0.94
Work experience in facility	6.38	6.88	7.85	8.74	0.00
Years since graduation	11.06	9.56	12.84	10.84	0.00
Any ANC training	0.46	0.50	0.46	0.50	0.90
Any child-health training	0.32	0.47	0.34	0.47	0.57
Any delivery training	0.39	0.49	0.42	0.49	0.25
Any newborn training	0.29	0.45	0.31	0.46	0.42
Number of relevant trainings	1.45	1.39	1.52	1.40	0.37
Any relevant training	0.64	0.48	0.66	0.47	0.39
Hours worked per week	44.11	17.24	47.33	16.64	0.00
N	447		2,604		

Note: Data are shown for providers observed during antenatal consultations in countries where both public and private facilities were sampled (DRC, Haiti, Kenya, Nepal, and Uganda).

Fig N. Structural quality by country in the private (for-profit) and public/non-profit (NP) sector

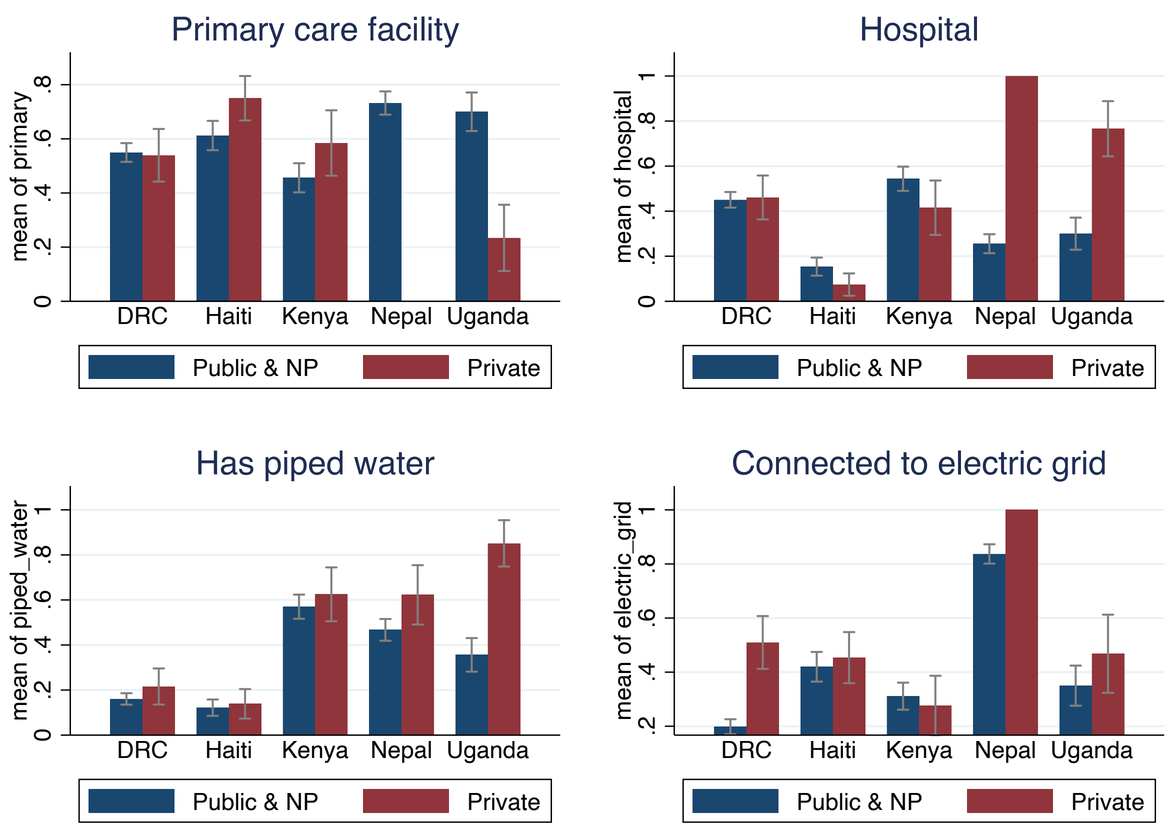

Note: The bar charts show unadjusted country-level means by sector with 95% confidence intervals.

Fig O. Structural quality by country in the private (for-profit) and public/non-profit (NP) sector

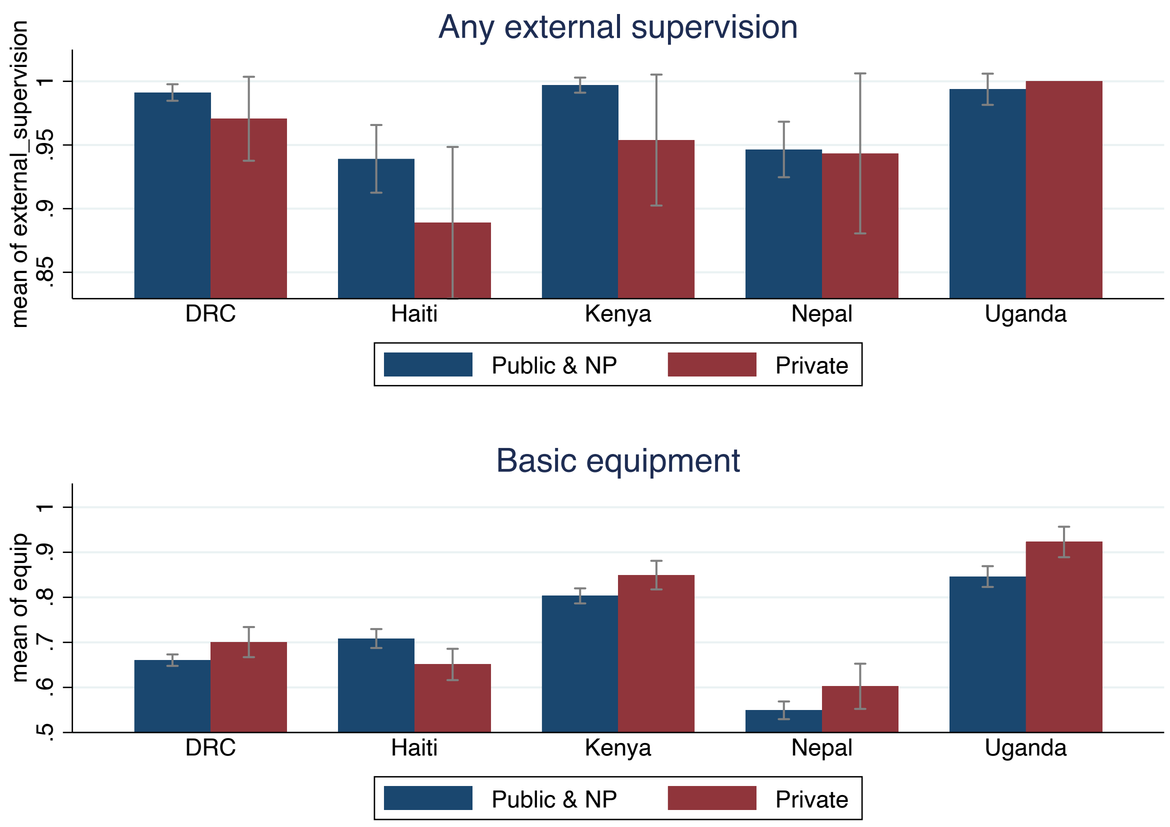

Note: The bar charts show unadjusted country-level means by sector with 95% confidence intervals.

Fig P. Provider characteristics by country in the private (for-profit) and public/non-profit (NP) sector

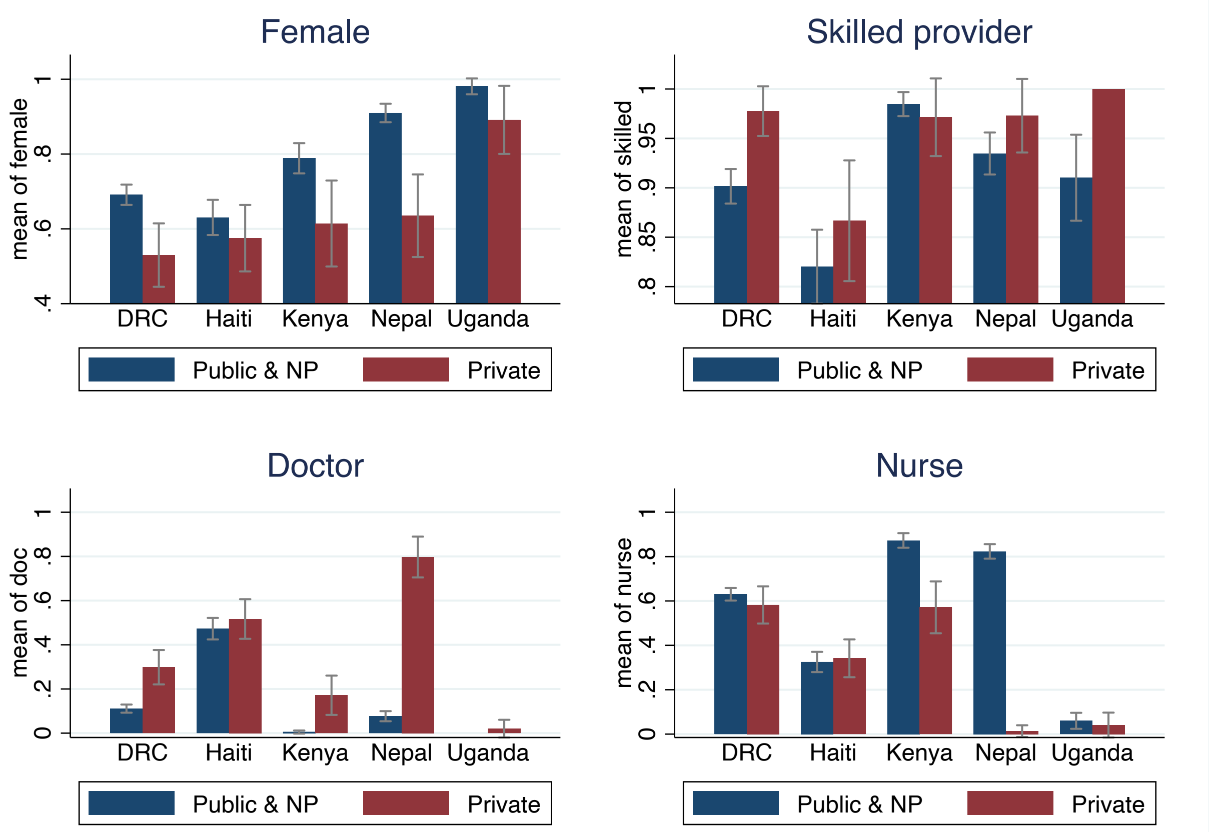

Note: The bar charts show unadjusted country-level means by sector with 95% confidence intervals.

Fig Q. Provider characteristics by country in the private (for-profit) and public/non-profit (NP) sector

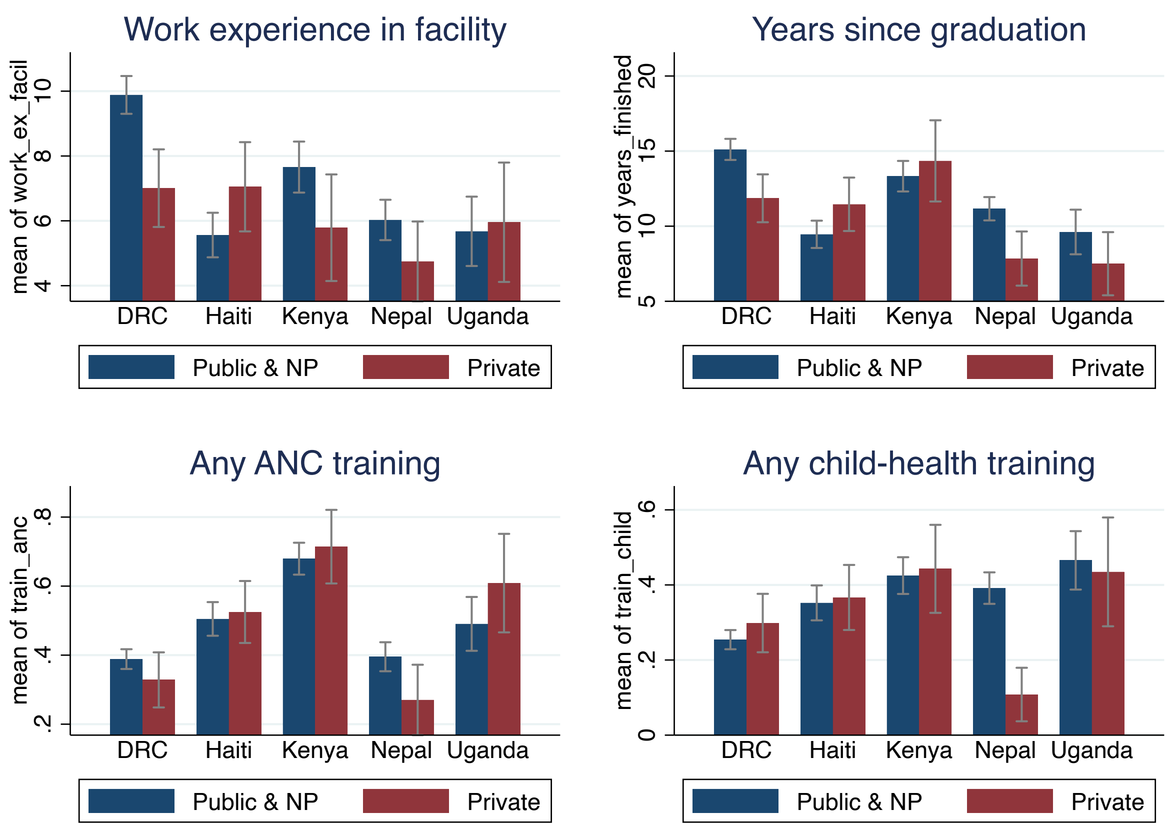

Note: The bar charts show unadjusted country-level means by sector with 95% confidence intervals.

Fig R. Provider characteristics by country in the private (for-profit) and public/non-profit (NP) sector

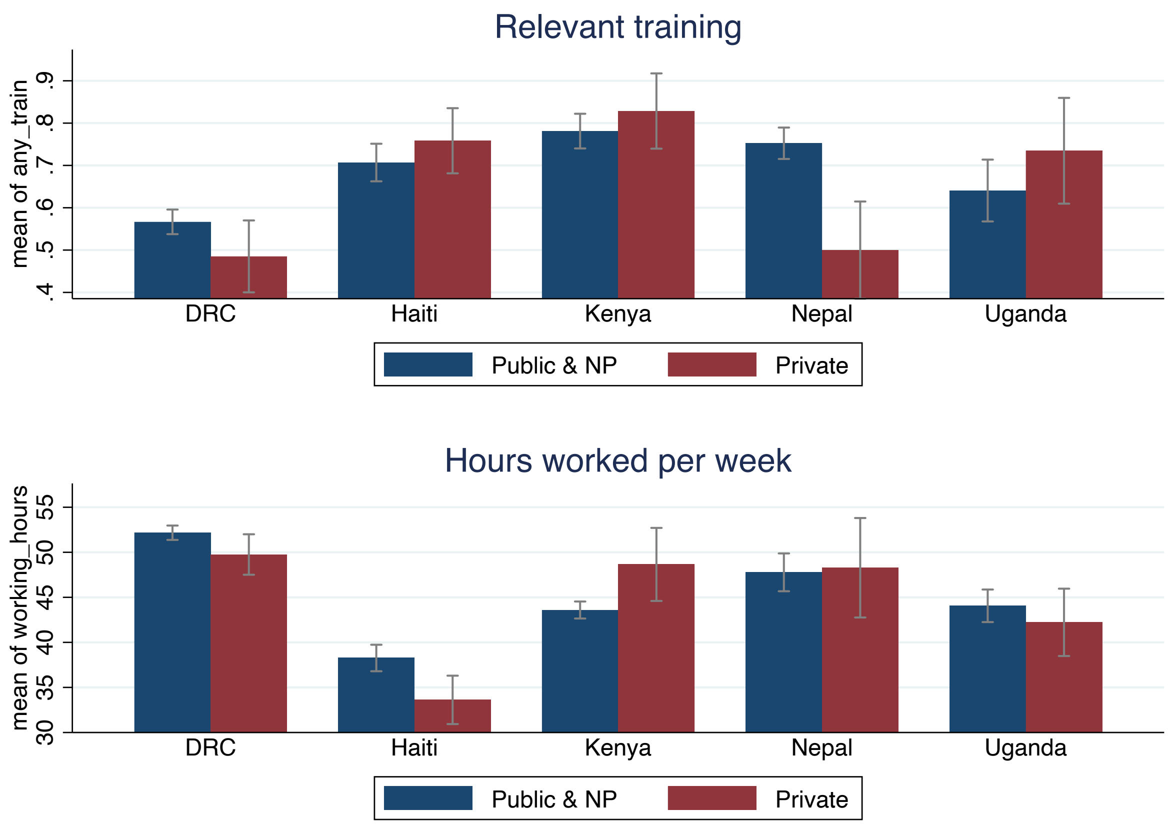

Note: The bar charts show unadjusted country-level means by sector with 95% confidence intervals.

Table X. Quality of healthcare in the private for-profit sector

VARIABLES	(1) Quality	(2) Quality	(3) Quality
Private for profit	0.011	0.009	0.011
	(0.011)	(0.010)	(0.010)
Observations	9,857	9,849	9,808
R-squared	0.414	0.493	0.496
Facility controls		✓	✓
Provider controls			✓

Note: All models include county fixed effects. Robust standard errors are in parentheses. Column 1 contains no control variables; column 2 adds controls for facility characteristics (level of care, access to water, electricity, supervision, availability of equipment); and column 3 additionally controls for provider characteristics available for all providers (gender, cadre). All models control for time of day and whether the consultation was a first visit.

Table Y. Quality of healthcare in the private for-profit sector (excluding non-profit organisations)

VARIABLES	(1) Quality	(2) Quality	(3) Quality
Private for profit	0.011	0.010	0.013
	(0.012)	(0.010)	(0.010)
Observations	7,755	7,747	7,706
R-squared	0.419	0.501	0.505
Facility controls		✓	✓
Provider controls			✓

Note: All models include county fixed effects. Robust standard errors are in parentheses. Column 1 contains no control variables; column 2 adds controls for facility characteristics; and column 3 additionally controls for provider characteristics.

Other determinants of clinical quality

Correlation with facility- and provider-level characteristics

To examine the correlation between quality of care and facility- and provider-level factors, we estimate the following OLS model:

$$Y_{ifpc} = \alpha F_f + \beta P_p + \eta_c + \epsilon_{ifpc}. \quad (2)$$

Shapley values are used to quantify the average marginal contribution of explanatory variables to the overall R^2 across all permutations of the order in which variables are entered, following (??).[5] To explore country-level differences, the model is estimated separately for each country. As sample sizes vary considerably across countries, we weight observations by $\frac{1{,}000}{N}$, where N is the number of observed consultations in a given country, reflecting that roughly 1,000 antenatal consultations were observed in most settings.

This section examines correlates of the quality of antenatal care in LMICs. Results are based on regressions of the form shown above. Column 1 in Table Z includes basic facility characteristics available for all countries. Column 2 adds information on whether facilities charge

[5] We use the `shapley2` command in Stata 17.

fees and on the number of staff (not available for Egypt). Column 3 adds basic provider characteristics (gender and cadre), and column 4 adds information on work experience and training (not available for 318 providers).

There is no evidence that the quality of care received by patients depends on the level of care: hospitals do not appear to provide higher quality than lower-level facilities. Quality is higher in facilities with better structural quality, particularly those with piped water (where providers complete around two percentage points more relevant actions). Availability of basic equipment is significantly correlated with quality in all specifications, although the magnitude is modest: a one standard-deviation increase in equipment availability is associated with a 0.7 percentage point increase in clinical quality. When all countries are pooled, there is no evidence that quality is higher in private for-profit facilities; estimates should be interpreted cautiously because some surveys sampled only public facilities. Pooled results indicate higher quality in facilities that charge fees; however, as discussed above, the amount paid is not positively correlated with quality. Overall, the facility-level variables included in the model explain 8.5% of the variance in quality.[6]

Columns 3 and 4 include provider-level variables. Skilled providers complete about four percentage points more relevant actions during antenatal consultations. There is no evidence of an association with work experience or training. Quality is around two percentage points higher for female providers, although female providers are not more likely to be skilled and do not appear to receive significantly more training. Provider-level factors explain 0.8% of the variance in quality of care.

Country fixed effects (included in all models but not shown) explain the largest share of variation in quality (39% of the variance).[7] This reflects large cross-country differences in quality. It is difficult to identify which mechanisms drive this variation because fixed effects capture differences in national income, health systems, and broader societal factors.

[6] Based on Shapley values.

[7] An additional three percent of the variance is attributable to controls for time of day and first-time visits (not shown).

Table Z. Quality of antenatal care and facility and provider characteristics

	Quality of antenatal care			
	(1)	(2)	(3)	(4)
Primary care facility	-0.004	-0.006	-0.001	-0.001
	(0.007)	(0.007)	(0.007)	(0.007)
Below primary care	-0.016	-0.006	0.011	0.011
	(0.016)	(0.017)	(0.017)	(0.017)
Has piped water	0.024***	0.018**	0.017**	0.018**
	(0.008)	(0.008)	(0.008)	(0.008)
Connected to electric grid	0.013*	0.012	0.009	0.009
	(0.007)	(0.007)	(0.007)	(0.007)
Any external supervision	-0.007	-0.013	-0.018	-0.017
	(0.015)	(0.015)	(0.015)	(0.016)
Basic equipment (std)	0.008**	0.008**	0.008**	0.007*
	(0.004)	(0.004)	(0.004)	(0.004)
Public	-0.011	-0.004	-0.004	-0.003
	(0.007)	(0.007)	(0.007)	(0.007)
Charges any fees		0.037***	0.041***	0.041***
		(0.014)	(0.013)	(0.014)
Nr. skilled medical staff		-0.000**	-0.000**	-0.000**
		(0.000)	(0.000)	(0.000)
Female			0.024***	0.023***
			(0.006)	(0.007)
Skilled provider			0.041***	0.043***
			(0.010)	(0.010)
Work experience in facility				0.000
				(0.000)
Relevant training				0.009
				(0.007)
Observations	21,434	20,372	20,326	20,008
R-squared	0.441	0.462	0.466	0.467

Note: OLS regressions. All models include county fixed effects, the weight $\frac{1,000}{N}$, and controls for first ANC visit and time of day (before noon). Robust standard errors clustered at facility level in parentheses. *** $p < 0.01$, ** $p < 0.05$, * $p < 0.1$.

The tables below reproduce the main analysis separately for each country. Results are broadly similar to the pooled analysis, with some exceptions. The positive association between quality and charging any fee does not hold in most settings and is negative in some. The correlation between quality and equipment availability is larger in Afghanistan (around four percentage points) and Tanzania (around two percentage points) than elsewhere. There is a significant correlation between quality and whether providers received any in-service training on maternal and neonatal health in Afghanistan (around five percentage points), DRC (around two percentage points), Kenya (around three percentage points), and Senegal (around eight percentage points). In Tanzania and Kenya, quality is around four percentage points higher in primary care facilities, whereas in Malawi quality is around four percentage points lower in primary care facilities. Finally, quality is significantly associated with supervision visits in the DRC (around ten percentage points) and Senegal (around seven percentage points). Even in the country-level analyses, most of the variation in quality remains unexplained (often 80% or more).

Table AA. Correlates of the quality of healthcare in Afghanistan

VARIABLES	(1)	(2)	(3)	(4)
Primary care facility	-0.015	0.002	0.002	-0.002
	(0.034)	(0.036)	(0.036)	(0.037)
Has piped water	-0.179***	-0.164***	-0.164***	-0.175***
	(0.035)	(0.037)	(0.037)	(0.036)
Connected to electric grid	-0.078*	-0.112**	-0.112**	-0.101**
	(0.042)	(0.043)	(0.043)	(0.041)
Any external supervision	0.006	-0.011	-0.011	-0.007
	(0.039)	(0.044)	(0.044)	(0.046)
Basic equipment (std)	-0.042**	-0.050***	-0.050***	-0.050***
	(0.018)	(0.017)	(0.017)	(0.016)
Public	-0.115***	-0.164***	-0.164***	-0.151***
	(0.036)	(0.053)	(0.053)	(0.055)
Charges any fees		0.032	0.032	0.047
		(0.057)	(0.057)	(0.053)
Number of skilled medical staff		0.000*	0.000*	0.000**
		(0.000)	(0.000)	(0.000)
Work experience in facility				0.000
				(0.003)
Relevant training				-0.040*
				(0.024)
Observations	494	494	494	494
R-squared	0.225	0.273	0.273	0.285

Note: OLS regressions. All models control for first ANC visit and time of day (before noon). Robust standard errors clustered at facility level in parentheses. *** $p < 0.01$, ** $p < 0.05$, * $p < 0.1$.

Table AB. Correlates of the quality of healthcare in the DRC

VARIABLES	(1)	(2)	(3)	(4)
Primary care facility	-0.002	-0.002	-0.000	-0.002
	(0.010)	(0.010)	(0.010)	(0.010)
Has piped water	0.018	0.016	0.014	0.014
	(0.011)	(0.012)	(0.012)	(0.012)
Connected to electric grid	0.024**	0.025**	0.023**	0.024**
	(0.011)	(0.011)	(0.011)	(0.011)
Any external supervision	0.098***	0.097***	0.100***	0.098***
	(0.025)	(0.025)	(0.025)	(0.027)
Basic equipment (std)	0.016**	0.016**	0.016**	0.016**
	(0.006)	(0.006)	(0.006)	(0.006)
Public	-0.007	-0.008	-0.006	-0.007
	(0.010)	(0.010)	(0.011)	(0.011)
Charges any fees		-0.121	-0.116	-0.116
		(0.096)	(0.096)	(0.095)
Number of skilled medical staff		0.000	0.000	0.000
		(0.000)	(0.000)	(0.000)
Female			0.001	0.002
			(0.009)	(0.009)
Skilled provider			0.045**	0.042**
			(0.018)	(0.019)
Work experience in facility				-0.000
				(0.000)
Relevant training				0.013
				(0.008)
Observations	4,517	4,517	4,517	4,511
R-squared	0.191	0.196	0.201	0.203

Note: OLS regressions. All models control for first ANC visit and time of day (before noon). Robust standard errors clustered at facility level in parentheses. *** $p < 0.01$, ** $p < 0.05$, * $p < 0.1$.

Table AC. Correlates of the quality of healthcare in Egypt

VARIABLES	(1)	(2)
Primary care facility	0.059**	0.062**
	(0.027)	(0.027)
Below primary care	0.057	0.063
	(0.039)	(0.041)
Has piped water	0.115***	0.117***
	(0.033)	(0.032)
Connected to electric grid	0.061**	0.061*
	(0.031)	(0.031)
Any external supervision	-0.005	0.002
	(0.053)	(0.051)
Basic equipment (std)	0.010	0.011
	(0.016)	(0.016)
Public	0.047	0.042
	(0.042)	(0.040)
Female		-0.009
		(0.021)
Skilled provider		0.051
		(0.054)
Observations	1,062	1,062
R-squared	0.116	0.119

Note: OLS regressions. All models control for first ANC visit and time of day (before noon). Robust standard errors clustered at facility level in parentheses. *** $p < 0.01$, ** $p < 0.05$, * $p < 0.1$.

Table AD. Correlates of the quality of healthcare in Ghana

VARIABLES	(1)	(2)	(3)	(4)
Primary care facility	-0.001	-0.007	-0.004	-0.021
	(0.021)	(0.022)	(0.021)	(0.022)
Has piped water	-0.011	-0.010	-0.010	-0.007
	(0.018)	(0.019)	(0.019)	(0.019)
Connected to electric grid	-0.002	-0.002	0.002	0.000
	(0.019)	(0.019)	(0.019)	(0.019)
Any external supervision	-0.014	-0.013	-0.013	-0.011
	(0.054)	(0.053)	(0.053)	(0.052)
Basic equipment (std)	0.018	0.019	0.015	0.016
	(0.012)	(0.012)	(0.013)	(0.013)
Public	0.022	0.028	0.025	0.033
	(0.020)	(0.022)	(0.022)	(0.021)
Charges any fees		0.002	0.001	0.002
		(0.029)	(0.027)	(0.028)
Number of skilled medical staff		-0.000	-0.000	-0.000
		(0.000)	(0.000)	(0.000)
Female			-0.028	-0.009
			(0.045)	(0.047)
Skilled provider			0.046**	0.048**
			(0.022)	(0.022)
Work experience in facility				-0.001
				(0.001)
Relevant training				0.009
				(0.016)
Observations	1,525	1,525	1,525	1,442
R-squared	0.050	0.053	0.059	0.084

Note: OLS regressions. All models control for first ANC visit and time of day (before noon). Robust standard errors clustered at facility level in parentheses. *** $p < 0.01$, ** $p < 0.05$, * $p < 0.1$.

Table AE. Correlates of the quality of healthcare in Haiti

VARIABLES	(1)	(2)	(3)	(4)
Primary care facility	-0.016	-0.021	-0.018	-0.019
	(0.016)	(0.017)	(0.017)	(0.017)
Below primary care	-0.032	-0.039*	-0.031	-0.031
	(0.021)	(0.022)	(0.023)	(0.024)
Has piped water	0.011	0.011	0.013	0.015
	(0.016)	(0.016)	(0.016)	(0.016)
Connected to electric grid	-0.018	-0.015	-0.016	-0.013
	(0.012)	(0.012)	(0.012)	(0.012)
Any external supervision	-0.011	-0.011	-0.016	-0.020
	(0.019)	(0.019)	(0.018)	(0.019)
Basic equipment (std)	0.008	0.008	0.008	0.008
	(0.006)	(0.006)	(0.006)	(0.006)
Public	0.009	0.011	0.010	0.007
	(0.013)	(0.013)	(0.013)	(0.013)
Charges any fees		0.022	0.027	0.031
		(0.033)	(0.032)	(0.031)
Number of skilled medical staff		-0.000	-0.000	-0.000
		(0.000)	(0.000)	(0.000)
Female			0.020**	0.018*
			(0.010)	(0.010)
Skilled provider			0.015	0.007
			(0.016)	(0.018)
Work experience in facility				-0.001
				(0.001)
Relevant training				0.005
				(0.011)
Observations	1,528	1,528	1,528	1,498
R-squared	0.106	0.108	0.114	0.113

Note: OLS regressions. All models control for first ANC visit and time of day (before noon). Robust standard errors clustered at facility level in parentheses. *** $p < 0.01$, ** $p < 0.05$, * $p < 0.1$.

Table AF. Correlates of the quality of healthcare in Kenya

VARIABLES	(1)	(2)	(3)	(4)
Primary care facility	0.014	0.037*	0.046**	0.045**
	(0.018)	(0.020)	(0.019)	(0.019)
Has piped water	0.043**	0.029*	0.028	0.026
	(0.017)	(0.017)	(0.017)	(0.017)
Connected to electric grid	-0.001	-0.001	0.006	0.003
	(0.017)	(0.017)	(0.016)	(0.016)
Any external supervision	-0.041	-0.064	-0.066	-0.059
	(0.077)	(0.069)	(0.051)	(0.047)
Basic equipment (std)	0.013	0.006	0.003	0.005
	(0.017)	(0.017)	(0.017)	(0.018)
Public	-0.011	-0.003	-0.007	-0.010
	(0.017)	(0.016)	(0.016)	(0.016)
Charges any fees		-0.083	-0.078	-0.086*
		(0.054)	(0.051)	(0.048)
Number of skilled medical staff		0.003***	0.003***	0.002***
		(0.001)	(0.001)	(0.001)
Female			0.052***	0.048***
			(0.016)	(0.016)
Skilled provider			0.147***	0.127***
			(0.032)	(0.033)
Work experience in facility				0.002*
				(0.001)
Relevant training				0.032*
				(0.018)
Observations	1,437	1,437	1,437	1,407
R-squared	0.166	0.191	0.215	0.230

Note: OLS regressions. All models control for first ANC visit and time of day (before noon). Robust standard errors clustered at facility level in parentheses. *** $p < 0.01$, ** $p < 0.05$, * $p < 0.1$.

Table AG. Correlates of the quality of healthcare in Malawi

VARIABLES	(1)	(2)	(3)	(4)
Primary care facility	-0.032*	-0.030	-0.029	-0.029
	(0.016)	(0.021)	(0.021)	(0.022)
Has piped water	0.030	0.029	0.026	0.020
	(0.022)	(0.022)	(0.022)	(0.022)
Connected to electric grid	0.008	0.007	0.005	0.004
	(0.014)	(0.015)	(0.015)	(0.015)
Any external supervision	0.027	0.030	0.031	0.024
	(0.037)	(0.038)	(0.038)	(0.043)
Basic equipment (std)	0.003	0.003	0.003	0.000
	(0.008)	(0.008)	(0.008)	(0.008)
Public	-0.017	-0.010	-0.010	-0.005
	(0.014)	(0.016)	(0.016)	(0.017)
Charges any fees		0.028	0.032	0.032
		(0.024)	(0.024)	(0.024)
Number of skilled medical staff		0.000	-0.000	0.000
		(0.004)	(0.004)	(0.004)
Female			0.016	0.004
			(0.014)	(0.014)
Skilled provider			0.016	0.041
			(0.027)	(0.028)
Work experience in facility				0.001
				(0.001)
Relevant training				0.015
				(0.018)
Observations	2,083	2,083	2,083	2,004
R-squared	0.091	0.093	0.097	0.099

Note: OLS regressions. All models control for first ANC visit and time of day (before noon). Robust standard errors clustered at facility level in parentheses. *** $p < 0.01$, ** $p < 0.05$, * $p < 0.1$.

Table AH. Correlates of the quality of healthcare in Namibia

VARIABLES	(1)	(2)	(3)	(4)
Primary care facility	-0.071	-0.074	-0.066	-0.016
	(0.066)	(0.067)	(0.065)	(0.050)
Has piped water	-0.009	-0.008	-0.001	0.012
	(0.052)	(0.052)	(0.050)	(0.051)
Connected to electric grid	-0.006	-0.007	-0.010	-0.005
	(0.022)	(0.022)	(0.022)	(0.021)
Any external supervision	-0.051	-0.060	-0.058	-0.066
	(0.052)	(0.054)	(0.052)	(0.057)
Basic equipment (std)	0.002	0.004	0.004	0.005
	(0.011)	(0.011)	(0.011)	(0.010)
Public	0.029	0.031	0.031	0.012
	(0.034)	(0.034)	(0.034)	(0.032)
Number of skilled medical staff		-0.000**	-0.000**	-0.000**
		(0.000)	(0.000)	(0.000)
Female			0.036	0.033
			(0.024)	(0.024)
Skilled provider			0.078***	0.099***
			(0.030)	(0.036)
Work experience in facility				-0.001
				(0.001)
Relevant training				-0.029
				(0.020)
Observations	825	825	825	813
R-squared	0.431	0.434	0.440	0.450

Note: OLS regressions. All models control for first ANC visit and time of day (before noon). Robust standard errors clustered at facility level in parentheses. *** $p < 0.01$, ** $p < 0.05$, * $p < 0.1$.

Table AI. Correlates of the quality of healthcare in Nepal

VARIABLES	(1)	(2)	(3)	(4)
Primary care facility	-0.013	-0.015	-0.015	-0.013
	(0.011)	(0.013)	(0.013)	(0.013)
Below primary care	0.039	0.038	0.040	0.040
	(0.044)	(0.045)	(0.044)	(0.042)
Has piped water	0.016*	0.017*	0.017*	0.019**
	(0.009)	(0.009)	(0.009)	(0.009)
Connected to electric grid	-0.003	-0.003	-0.003	-0.004
	(0.013)	(0.013)	(0.013)	(0.014)
Any external supervision	-0.013	-0.013	-0.013	0.000
	(0.022)	(0.020)	(0.020)	(0.021)
Basic equipment (std)	0.004	0.004	0.004	0.002
	(0.005)	(0.005)	(0.005)	(0.005)
Public	-0.014	-0.014	-0.015	-0.014
	(0.013)	(0.013)	(0.013)	(0.014)
Charges any fees		-0.004	-0.003	0.001
		(0.011)	(0.011)	(0.011)
Number of skilled medical staff		-0.000	0.000	0.000
		(0.001)	(0.001)	(0.001)
Female			0.007	0.003
			(0.013)	(0.014)
Skilled provider			-0.014	-0.016
			(0.020)	(0.020)
Work experience in facility				-0.000
				(0.001)
Relevant training				0.008
				(0.012)
Observations	1,565	1,565	1,565	1,508
R-squared	0.069	0.069	0.070	0.069

Note: OLS regressions. All models control for first ANC visit and time of day (before noon). Robust standard errors clustered at facility level in parentheses. *** $p < 0.01$, ** $p < 0.05$, * $p < 0.1$.

Table AJ. Correlates of the quality of healthcare in Rwanda

VARIABLES	(1)	(2)	(3)	(4)
Primary care facility	0.199***	0.027	0.023	-0.004
	(0.063)	(0.081)	(0.082)	(0.084)
Has piped water	0.029	0.029	0.030	0.029
	(0.030)	(0.030)	(0.031)	(0.031)
Connected to electric grid	0.049	0.059*	0.059*	0.065**
	(0.031)	(0.031)	(0.032)	(0.032)
Any external supervision	0.051	0.053	0.037	0.052
	(0.073)	(0.068)	(0.080)	(0.080)
Basic equipment (std)	0.041**	0.041*	0.040*	0.037*
	(0.021)	(0.021)	(0.021)	(0.021)
Public	0.059	0.058	0.060	0.054
	(0.036)	(0.037)	(0.037)	(0.037)
Charges any fees		-0.211***	-0.204***	-0.228***
		(0.042)	(0.040)	(0.044)
Number of skilled medical staff		-0.001**	-0.001*	-0.001*
		(0.001)	(0.001)	(0.001)
Female			0.032	0.034
			(0.047)	(0.050)
Skilled provider			0.003	-0.017
			(0.038)	(0.049)
Work experience in facility				-0.003
				(0.003)
Relevant training				0.052
				(0.032)
Observations	737	737	732	727
R-squared	0.142	0.152	0.155	0.172

Note: OLS regressions. All models control for first ANC visit and time of day (before noon). Robust standard errors clustered at facility level in parentheses. *** $p < 0.01$, ** $p < 0.05$, * $p < 0.1$.

Table AK. Correlates of the quality of healthcare in Senegal

VARIABLES	(1)	(2)	(3)	(4)
Primary care facility	0.048	0.046	0.051	0.046
	(0.032)	(0.039)	(0.040)	(0.040)
Has piped water	-0.010	-0.009	-0.008	-0.003
	(0.033)	(0.033)	(0.032)	(0.032)
Connected to electric grid	0.060***	0.060***	0.046**	0.055**
	(0.023)	(0.023)	(0.022)	(0.023)
Any external supervision	0.052**	0.045*	0.056**	0.060**
	(0.021)	(0.024)	(0.028)	(0.030)
Basic equipment (std)	0.011	0.011	0.013	0.010
	(0.011)	(0.011)	(0.011)	(0.011)
Public	-0.006	-0.005	-0.003	-0.005
	(0.031)	(0.031)	(0.031)	(0.031)
Charges any fees		0.030	0.009	-0.004
		(0.035)	(0.034)	(0.038)
Number of skilled medical staff		-0.000	-0.000	0.000
		(0.001)	(0.001)	(0.001)
Female			0.128***	0.112***
			(0.031)	(0.034)
Skilled provider			-0.036	-0.037
			(0.032)	(0.032)
Work experience in facility				-0.003**
				(0.001)
Relevant training				0.090***
				(0.024)
Observations	849	849	849	849
R-squared	0.125	0.125	0.154	0.173

Note: OLS regressions. All models control for first ANC visit and time of day (before noon). Robust standard errors clustered at facility level in parentheses. *** $p < 0.01$, ** $p < 0.05$, * $p < 0.1$.

Table AL. Correlates of the quality of healthcare in Tanzania

VARIABLES	(1)	(2)	(3)	(4)
Primary care facility	-0.027	0.018	0.022	0.020
	(0.018)	(0.021)	(0.021)	(0.022)
Has piped water	0.018	0.019	0.017	0.018
	(0.016)	(0.016)	(0.016)	(0.016)
Connected to electric grid	0.026	0.024	0.010	0.010
	(0.016)	(0.017)	(0.017)	(0.017)
Any external supervision	0.004	0.013	0.030	0.031
	(0.055)	(0.062)	(0.058)	(0.058)
Basic equipment (std)	0.032***	0.031***	0.028***	0.028***
	(0.007)	(0.007)	(0.006)	(0.006)
Public	0.054***	0.050***	0.043**	0.043***
	(0.017)	(0.017)	(0.017)	(0.017)
Charges any fees		0.048*	0.036	0.036
		(0.027)	(0.026)	(0.026)
Number of skilled medical staff		0.005***	0.004**	0.004**
		(0.001)	(0.002)	(0.002)
Female			0.005	0.006
			(0.020)	(0.020)
Skilled provider			0.097***	0.097***
			(0.016)	(0.016)
Work experience in facility				-0.000
				(0.001)
Relevant training				-0.002
				(0.015)
Observations	4,010	4,010	4,010	3,996
R-squared	0.148	0.156	0.179	0.180

Note: OLS regressions. All models control for first ANC visit and time of day (before noon). Robust standard errors clustered at facility level in parentheses. *** $p < 0.01$, ** $p < 0.05$, * $p < 0.1$.

Table AM. Correlates of the quality of healthcare in Uganda

VARIABLES	(1)	(2)	(3)	(4)
Primary care facility	-0.044	-0.040	-0.001	0.006
	(0.042)	(0.047)	(0.044)	(0.045)
Has piped water	-0.006	-0.008	-0.006	0.001
	(0.044)	(0.043)	(0.040)	(0.041)
Connected to electric grid	0.007	0.007	0.003	-0.001
	(0.031)	(0.031)	(0.030)	(0.031)
Basic equipment (std)	0.018	0.017	0.005	0.009
	(0.022)	(0.022)	(0.024)	(0.024)
Public	-0.021	-0.012	-0.013	-0.017
	(0.034)	(0.045)	(0.046)	(0.042)
Charges any fees		0.015	0.032	0.025
		(0.049)	(0.050)	(0.047)
Number of skilled medical staff		0.000	0.001	0.001
		(0.000)	(0.001)	(0.001)
Female			0.052	0.049
			(0.083)	(0.076)
Skilled provider			0.073	0.065
			(0.047)	(0.047)
Work experience in facility				0.003
				(0.002)
Relevant training				-0.001
				(0.028)
Any external supervision	0.136***	0.135***		
	(0.029)	(0.030)		
Observations	802	802	761	759
R-squared	0.203	0.203	0.217	0.224

Note: OLS regressions. All models control for first ANC visit and time of day (before noon). Robust standard errors clustered at facility level in parentheses. *** $p < 0.01$, ** $p < 0.05$, * $p < 0.1$.

Quality of care and amount paid during the consultation

To estimate consultation fees, we use data from patient exit interviews, where patients report the total amount paid during the consultation in local currency. The analysis cannot distinguish between facility-level prices and differences in the volume of services rendered. To provide an approximate cross-country comparison, the amount paid in each country is converted into international dollars using the PPP conversion factor for private consumption for the relevant year.[8] Because this approach does not capture subnational variation in purchasing power, analyses of consultation fees are conducted at the country level.

This section examines whether women who pay more for antenatal consultations receive higher clinical quality of care, given substantial within-country variation in payments. Because women report only the total amount paid, variation may reflect both differences in prices for the same services and differences in the volume of services delivered. In this setting, however, there is no mechanical link between the clinical quality measure and prices: required actions during antenatal consultations are primarily based on provider effort (questions asked and basic examinations) and do not inherently require expensive tests or procedures. Moreover, there are no expensive procedures that are close substitutes for provider effort, which is the focus of the clinical quality measure used here.[9]

Table AN summarises fees paid in the relevant countries, indicating substantial within-country variation. Cross-country differences should be interpreted cautiously because international dollars are a simplified approach to capturing purchasing power. These values should not be interpreted relative to GDP per capita in nominal US dollars.

Table AO reports the association between clinical quality and a one standard-deviation increase in the amount paid. Results are shown separately by country to reflect differences in purchasing power that cannot be fully accounted for. Column 1 includes all countries with data on user fees (excluding Egypt, Ghana, and Uganda), while columns 2 and 3 show estimates for countries where both public and private facilities were sampled. Overall, there is little evidence of a strong link between how much women pay and the quality of care received. In the DRC, a one standard-deviation increase in fees is associated with a two percentage point increase in quality in private for-profit facilities; a similar pattern is observed in Nepal (in both public and private for-profit facilities). In Senegal, where the

[8] PPP conversion factor for private consumption: https://data.worldbank.org/indicator/PA.NUS.PRVT.PP.

[9] The quality measure treats ultrasound as a substitute for palpating the abdomen and listening for the fetal heartbeat.

data cover almost exclusively public facilities, a one standard-deviation increase in fees is associated with a two percentage point decrease in quality. In other settings, there is no statistically significant association. Hence, variation in what women pay for antenatal care does not, in general, reflect differences in clinical quality.

Table AN. Quality of healthcare and the amount paid

	Quality and fees paid		
	All	Public	Private
	(1)	(2)	(3)
DRC (2017)	-0.001	0.010*	0.024***
	(0.010)	(0.006)	(0.008)
Haiti (2018)	-0.017*	-0.011	0.000
	(0.009)	(0.069)	(0.001)
Kenya (2010)	-0.016	0.071	-0.000
	(0.010)	(0.075)	(0.004)
Nepal (2015)	-0.001	0.023**	0.016**
	(0.011)	(0.009)	(0.008)
Afghanistan (2018)	0.000		
	(0.000)		
Malawi (2013)	-0.011		
	(0.011)		
Namibia (2009)	-0.027		
	(0.026)		
Senegal (2016)	-0.023**		
	(0.011)		
Tanzania (2014)	0.001		
	(0.015)		
Observations	7,153	4,566	1,058
R-squared	0.359	0.471	0.280

Note: OLS regressions. Coefficients report the change in quality associated with a one standard-deviation increase in the amount paid. Models include an interaction between user fees and country. All models control for facility and provider characteristics (public facility, primary care, access to water, access to electricity, supervision, availability of basic equipment, gender, cadre). Robust standard errors clustered at facility level in parentheses. *** $p < 0.01$, ** $p < 0.05$, * $p < 0.1$.

Table AO. Amount paid during the consultation (international dollars)

	Obs	Mean	Std. dev.	25th pct.	75th pct.
Afghanistan	302	50.31	61.24	20.53	58.64
DRC	2,764	6.23	10.33	1.59	6.34
Haiti	1,117	8.43	74.88	1.46	6.30
Kenya	888	6.15	25.96	0.62	5.28
Malawi	244	10.61	28.02	3.40	9.34
Namibia	45	1.26	0.97	0.80	1.60
Nepal	556	13.53	21.34	1.00	16.71
Rwanda	153	2.69	8.07	0.25	1.48
Senegal	775	5.14	7.03	2.11	5.91
Tanzania	471	12.51	38.03	2.12	11.32

Note: Values are expressed in international dollars (PPP-adjusted) using the PPP conversion factor for private consumption. International dollars are intended to equalise purchasing power across countries; however, for low-income economies these values can appear large relative to those in higher-income countries and should be interpreted cautiously.
