# Supplementary material for: Clinical process quality of antenatal care in low and middle-income countries: Cross-sectional evidence from 13 countries
Source: PLOS Glob Public Health. 2026 Feb 24;6(2):e0005990. doi: 10.1371/journal.pgph.0005990 (PMC12931779; doi:10.1371/journal.pgph.0005990)
Supplement: Attachment — Submitted filename: Response to Reviewers.docx [file pgph.0005990.s003.docx]

**Response to Reviewers**

Title: Clinical process quality of antenatal care in low and middle-income countries: Cross-sectional evidence from 13 countries
Reference: PLOS Global Public Health (PGPH-D-25-02697)

I thank the Editor and the reviewers for their careful reading of the manuscript and for the constructive comments provided. I have revised the manuscript to address all points raised. Below, I respond to each comment in turn, using blue text, and indicate how the manuscript has been amended.

**Reviewer #1:**

This manuscript addresses an important and highly relevant public health question: the quality of clinical processes during antenatal care (ANC) in low- and middle-income countries (LMICs), and differences between public and private-for-profit providers. Using a large, multi-country dataset covering 21,850 ANC consultations across 13 LMICs, the study makes a valuable contribution to the literature by focusing on observed provider actions rather than service utilization alone. Overall, the manuscript is methodologically sound, the analyses are appropriate, and the conclusions are generally well supported by the data. I recommend minor revisions to improve clarity, structure, and interpretability.

Below are specific comments organized by manuscript section.

Abstract
The abstract is clear and informative; however, it could be strengthened by briefly clarifying how “clinical process quality” is operationalized (e.g., proportion of recommended history questions, examinations, tests, and treatments performed).

This has been added.

Consider adding a short phrase highlighting the policy relevance of comparing public versus private-for-profit facilities, particularly in resource-constrained settings.

We have added this clarification to the manuscript

Introduction
The introduction provides strong global context regarding maternal mortality and the importance of antenatal care. However, the flow could be improved for clarity and coherence.

The introduction has been edited to improve flow and clarity.

Consider more explicitly defining the link between poor-quality antenatal care and maternal and neonatal outcomes, potentially supported by a small number of additional references.

The link has been made more explicit and additional references have been added:

- However, a growing body of evidence shows that increased utilisation of maternal health services does not, by itself, improve maternal and neonatal outcomes when the quality of antenatal and intrapartum care is poor. Low-quality antenatal care limits effective screening and management of conditions such as hypertensive disorders, anaemia, and infections, and reduces opportunities for timely referral and evidence-based preventive interventions, all of which are key pathways linking care quality to maternal and neonatal morbidity and mortality4.

The discussion of LMICs could be expanded slightly to clarify the structural and systemic challenges affecting care quality (e.g., workforce constraints, accountability mechanisms, resource limitations).

This has been expanded:

- This study provides the broadest overview to date of the clinical process quality of antenatal care in LMICs and presents data on the actions taken by healthcare providers in 21,850 consultations in 13 LMICs (…), **settings characterised by substantial workforce, resource, and governance constraints that may affect care quality4–7.**

While the rationale for comparing public and private providers is well articulated, the introduction would benefit from a clearer transition that explicitly links maternal mortality, ANC quality, and provider incentives.

The following has been added to make this link clearer:

- Using a large multi-country dataset, this study also speaks to one of the key policy issues in healthcare provision in the world’s poorest countries: whether care is best provided by the public or the private sector. Given the central role of antenatal care in identifying and managing pregnancy-related risks that contribute to maternal and neonatal mortality, differences in provider incentives across sectors may have important implications for the quality of care delivered during routine ANC consultations.

The number of references is acceptable; however, adding a few targeted citations (particularly on ANC quality and provider performance in LMICs) could strengthen the theoretical framing. This is a suggestion rather than a requirement.

We have added further references focused on ANC quality, including Kruk et al. (2018) and Benova et al. (2021), to strengthen the framing.

Methods
The data sources, sampling strategy, and inclusion criteria are clearly described and appropriate for the research question.
The construction of the main outcome variable (proportion of relevant clinical actions

performed) is reasonable and well motivated. A brief justification for weighting all actions equally (if applicable) could further strengthen this section.

This has been clarified, items are equally weighted:

- The main outcome used in the analysis is the clinical process quality of care provided, defined as the proportion of essential history questions asked, physical examinations conducted, advice given, and preventive tests and treatments recommended or prescribed. Each action is weighted equally, reflecting their joint role as non-substitutable components of guideline-recommended antenatal care. The measure captures actions that should be completed at each antenatal visit, accounting for gestational age and whether the consultation is a first or follow-up visit.

The classification of facilities into public and private-for-profit is clear; however, a short discussion acknowledging heterogeneity within these categories would improve transparency.

This has been made clearer in the manuscript:

- We distinguish between public and private-for-profit facilities. While this classification captures an important institutional distinction relevant for policy, it necessarily aggregates heterogeneous providers within each category, including differences in ownership structures, management practices, and contractual arrangements. As a result, estimates should be interpreted as average differences across broad sectors rather than as comparisons between uniform types of facilities.

The statistical approach is appropriate. Minor clarifications regarding model specification (e.g., choice of controls, fixed effects) would enhance reproducibility, but no major methodological concerns were identified.

Specific controls has been clarified in the revised text to improve clarity.

Results
Results are presented clearly and logically, with appropriate use of tables and figures.
The description of cross-country variation in ANC quality is informative and highlights important heterogeneity. When discussing differences between public and private-for-profit facilities, it may be helpful to more explicitly emphasize effect sizes and their practical significance, not only statistical significance.

We have added coefficient estimates to help interpretation:

- There is no evidence to suggest that private-for-profit facilities provide higher quality care in any of the study countries. Coefficients are small in magnitude and not significant (coefficient estimates are 0.00 for Haiti, -0.03 for Kenya, 0.013 for Nepal and 0.06 for Uganda).

Consider briefly restating the key findings at the end of the Results section to aid reader comprehension.

We have added a brief summary statement at the end of the section:

- Taken together, the results show that the clinical process quality of antenatal care in LMICs is low and highly variable within and across countries, and that this variation is poorly explained by commonly used structural proxies such as infrastructure, equipment, staffing, or prices. Importantly, there is no evidence to suggest that private for-profit facilities provide higher-quality antenatal care than public facilities, despite being better resourced and substantially more costly on average.

Discussion
The discussion appropriately interprets the findings and avoids overstatement.
The policy implications are relevant and well aligned with the study results; however, the discussion could be strengthened by more explicitly linking findings to potential health system interventions (e.g., provider incentives, supervision, quality monitoring).

The discussion has been expanded to link findings with policies more explicitly:

- First, the clinical process quality of healthcare is critically low in many LMICs. To reach key policy targets around women’s health and maternal mortality, clinical process quality needs to improve. However, existing evidence provides limited and inconclusive guidance on how best to achieve sustained improvements in provider performance in routine care settings. While a wide range of intervention – such as in-service training, supervision, audit and feedback, and incentives – have been implemented, systematic reviews suggest that their effects on clinical practice are often modest, highly context-specific, and difficult to sustain at scale23,24. As a result, improving clinical process quality remains a major unresolved challenge in health systems in LMICs.

The limitations section is appropriate, though it could be expanded slightly to emphasize issues such as unobserved provider characteristics or potential measurement limitations of process quality indicators.

The section has been amended to emphasise both of these points:

- This study is limited in a number of respects. First, estimates of the quality of antenatal care are subject to an observation bias. Hence, even though average levels of quality of care are very low, these estimates likely present upper bounds. **In addition, the analysis cannot fully account for unobserved provider characteristics – such as intrinsic motivation, experience, or clinical knowledge – that may influence performance and contribute to residual variation in measured quality.** … Finally, we only focus on the clinical quality of care and disregard other less technical aspects of the quality of services – such as provider demeanour or bedside manner, as well as outcome indicators such as mortality. **Process quality indicators also capture adherence to guideline-recommended actions but may not fully reflect appropriate clinical judgement or case-specific decision-making in all circumstances.** Nonetheless, the clinical quality of care plays a key role in determining the degree to which uptake of antenatal care influences patient outcomes and is therefore paramount for reaching international development targets around maternal and neonatal health.

The conclusion is supported by the data and appropriately cautious.

Presentation and Language

The manuscript is written in clear, standard English and is generally well organized.
Minor typographical and formatting issues are present and should be addressed during revision, but these do not detract from the scientific content.

Overall Assessment

This is a strong and policy-relevant manuscript that makes a meaningful contribution to the literature on maternal health and healthcare quality in LMICs. The suggested revisions are primarily focused on improving clarity, structure, and contextualization rather than addressing substantive methodological concerns. I therefore recommend acceptance pending minor revisions.

**Reviewer #2:**

Great analysis. Nothing to add. Absolutely interesting topic especially for 13 countries included in the study. Antenatal care unfortunately, still is big problem for low and some of the middle income countries all over the world. You address this problem clearly though meticulously analysing multiple factors. So conclusions are clear.
